# Assessing Renal Outcomes After Switching from Tenofovir Disoproxil Fumarate to Tenofovir Alafenamide in People with HIV: Proteinuria Changes and Limits of Causal Attribution

**DOI:** 10.3390/diseases14100353

**Published:** 2026-09-24

**Authors:** Tommaso D’Anna, Marcello Trizzino, Beatrice Belmonte, Claudia Gioè, Augusto Orlandi, Stefania Zerbo, Andrea Cicero, Antonina Argo, Antonio Cascio

**Affiliations:** 1 Department of Biomedicine and Prevention, Tor Vergata University of Rome, Via Montpellier 1, 00133 Rome, Italy; orlandi@uniroma2.it; 2 Tumor Immunology Unit, Department of Health Promotion, Mother and Child Care, Internal Medicine and Medical Specialties (PROMISE), University of Palermo, Corso Tukory 211, 90134 Palermo, Italy; beatrice.belmonte@unipa.it; 3 Legal Medicine Unit, University Hospital “Paolo Giaccone”, Via del Vespro 129, 90127 Palermo, Italy; stefania.zerbo@unipa.it (S.Z.); antonella.argo@unipa.it (A.A.); 4 Infectious and Tropical Diseases Unit, University Hospital “Paolo Giaccone”, Via del Vespro 129, 90127 Palermo, Italy; marcello.trizzino@policlinico.pa.it (M.T.); claudia.gioe@policlinico.pa.it (C.G.); antonio.cascio03@unipa.it (A.C.); 5 Department of Health Promotion, Mother and Child Care, Internal Medicine and Medical Specialties (PROMISE), University of Palermo, Piazza delle Cliniche, 2, 90127 Palermo, Italy; andrea.cicero@community.unipa.it

**Keywords:** tenofovir disoproxil fumarate, tenofovir alafenamide, HIV, proteinuria, urinary protein-to-creatinine ratio, renal function, prediction modelling, causal attribution, retrospective cohort

## Abstract

**Background:** The total urinary protein-to-creatinine ratio (PCr) measures proteinuria but does not establish tubular injury or its recovery. **Methods:** We examined PCr changes after switching from tenofovir disoproxil fumarate (TDF) to tenofovir alafenamide (TAF) in a single-centre retrospective cohort of 364 adults with human immunodeficiency virus (HIV) infection. Paired PCr values were available for 159 patients (43.7%). The primary descriptive endpoint was a PCr reduction below 20%, an operational, nonvalidated threshold. Analyses comprised paired rank-based comparisons, baseline-adjusted regression, threshold and influence sensitivity analyses, and nested cross-validation of ridge logistic regression using three predictor sets. **Results:** The median paired PCr change was −4.0 mg/g (interquartile range −41.5 to 11.5; signed-rank Benjamini–Hochberg-adjusted q = 0.0098); 99/159 patients (62.3%) had a reduction below 20%. Baseline log(1 + PCr) was positively associated with post-switch log(1 + PCr), while its coefficient in the corresponding change model was exactly one unit lower, illustrating mathematical coupling. The cross-validated area under the curve was 0.709 for baseline PCr alone, 0.475 for the other covariates, and 0.701 for all predictors. **Conclusions:** PCr changes were heterogeneous, and additional covariates did not improve discrimination over the baseline PCr alone. Missing outcomes, unavailable specimen dates, the absence of tubular-specific biomarkers and the uncontrolled design preclude conclusions about irreversible tubular injury, causal treatment effects or clinical prediction utility.

## 1. Introduction

Tenofovir disoproxil fumarate (TDF) has been a cornerstone of antiretroviral therapy (ART) because of its potent activity against human immunodeficiency virus (HIV) and hepatitis B virus (HBV), high genetic barrier in combination regimens and suitability for once-daily fixed-dose treatment. However, long-term exposure has been associated with proximal tubular dysfunction, proteinuria, a declining estimated glomerular filtration rate (eGFR), Fanconi syndrome, phosphate wasting and bone mineral loss, making renal and skeletal safety central to contemporary HIV pharmacotherapy [1,2,3].

TDF nephrotoxicity is primarily due to systemic tenofovir exposure following plasma hydrolysis. Tenofovir is filtered and actively handled by proximal tubular epithelial cells, where basolateral uptake is mediated by organic anion transporters 1 and 3 (OAT1 and OAT3), while apical efflux depends on multidrug resistance-associated protein 4 (MRP4). Transporter-dependent intracellular accumulation promotes mitochondrial injury and tubular dysfunction [4]. This mechanism is consistent with experimental evidence of proximal tubular mitochondrial damage and altered mitochondrial deoxyribonucleic acid (DNA) homeostasis [5] and explains the clinical phenotype of tubular proteinuria, normoglycaemic glycosuria, phosphaturia, hypophosphataemia, renal tubular acidosis and Fanconi syndrome.

Renal tubular toxicity may coexist with disturbances of phosphate handling and bone metabolism [2]. Large cohort data have associated longer tenofovir exposure with proteinuria and declining renal function [6]. Tenofovir alafenamide (TAF) was developed to reduce systemic tenofovir exposure while maintaining antiviral efficacy [7]. Comparative trials and switch studies have reported favourable renal safety profiles and changes in proteinuria, tubular markers and bone mineral density (BMD) [8,9,10]. These outcomes must be distinguished from recovery of established structural kidney injury in an individual patient.

Nevertheless, favourable average renal safety findings do not imply complete or uniform improvement in every patient. Cohort studies have reported changes in eGFR and tubular markers after TDF discontinuation or TDF-to-TAF switching, with the observed course varying by baseline renal function and the outcome definition [11,12,13,14]. These studies support investigating heterogeneous laboratory trajectories, while recognising that neither total proteinuria nor eGFR alone identifies the mechanism of kidney injury.

Urinary biomarkers differ in their biological specificity. The total urinary protein-to-creatinine ratio (PCr) measures proteinuria arising from glomerular, tubular or mixed processes; it cannot, by itself, establish proximal tubular injury or its resolution. Low-molecular-weight proteins, including retinol-binding protein (RBP) and β2-microglobulin (β2M), provide more specific information about proximal tubular handling [15,16,17]. Routine clinical datasets often contain serum creatinine, estimated glomerular filtration rate (eGFR) and total PCr without these additional markers. Such datasets can characterise changes in proteinuria, but cannot distinguish recovery from established tubulopathy from other causes of change.

Recent observational cohorts provide longer-term renal follow-up after TAF initiation or TDF-to-TAF switching, but changes in total proteinuria, tubular markers and creatinine-based eGFR do not necessarily track together [18,19,20]. This supports a focused assessment of the particular marker and endpoint available in routine clinical data.

We therefore examined the distribution and heterogeneity of PCr changes in a retrospective cohort of people with HIV who switched from TDF to TAF. The primary descriptive endpoint was a PCr reduction of less than 20%, an operational definition retained from the original analysis rather than a validated threshold for renal recovery. A separate exploratory prediction analysis asked whether routinely available covariates added information beyond baseline PCr alone. Because baseline PCr also enters the endpoint formula, we explicitly examined mathematical coupling and regression to the mean [21]. The study was not designed to establish that TAF caused the observed changes, to diagnose irreversible tubular injury, or to develop a clinically deployable prediction tool. Distinguishing documented biochemical change from renal injury and its attribution to a specific exposure is central to interpreting suspected treatment-related renal harm.

## 2. Materials and Methods

### 2.1. Study Design and Participants

This single-centre retrospective observational study used routine-care records from the Infectious and Tropical Diseases Unit of the University Hospital “Paolo Giaccone” of Palermo, Italy. The clinical inclusion criteria were adult HIV infection, previous TDF-containing ART and a subsequent switch to a TAF-containing regimen. Data abstraction was conducted from 1 January to 31 May 2026. Switch status was confirmed for all 364 reconstructed patients, including those without a recorded switch date. A missing laboratory result did not exclude a patient from the cohort; each analysis used its explicitly defined evaluable subset. No intervention, randomisation or additional patient contact was undertaken.

The source contained 391 treatment records. Twenty patients had repeated records (16 with two, three with three and one with six), giving 27 excess rows. These were not exact duplicates: the recorded TDF treatment dates differed in all 20 groups, and laboratory values differed in 18. One row per patient was retained using the original rule of maximum nonmissing-field completeness, with the source row order breaking ties; seven groups had a completeness tie. One clinically populated record lacked a usable source identifier and was assigned an anonymous study key after checking that its identifying information did not duplicate another source record. These steps yielded 364 patient-level records. The selected TDF episode is not necessarily lifetime cumulative exposure, and completeness-based selection may favour better documented episodes.

One erroneous date of birth generated an apparent age of 8 years; this was confirmed as a data entry error and treated as missing, without inferring a replacement birth date. Age at switch was derived from valid birth and TAF start dates and was available for 254 patients. The primary PCr endpoint required both phase measurements and a strictly positive baseline PCr; all 159 evaluable patients had a recorded switch date.

### 2.2. Data Collection

Clinical and laboratory data were extracted from routine outpatient records into a structured spreadsheet. Available fields included demographic information, date of first HIV contact, known duration of HIV infection, HBV and hepatitis C virus (HCV) coinfection, recorded previous renal disease, TDF treatment dates and duration, protease inhibitor (PI) exposure, TAF start dates and discontinuation indicators, regimen and follow-up indicators, renal markers and bone mineral measurements. Original column names and values were retained in the restricted source file; analytical variables were standardised in a separate reproducible workflow. Source identifiers were used only for patient reconstruction. The shared data package provides de-identified versions of the 391-record source workbook and 364-patient numeric reference, together with the analytical dataset and code. Names, original identifiers, birth dates, calendar dates and free-text regimens are excluded; random study keys, relative day offsets and age at switch in days preserve the quantities needed for reproduction. The README documents these transformations and the reconstruction rule.

The analytical source did not contain cluster of differentiation 4 (CD4) cell counts, plasma HIV ribonucleic acid (RNA), HIV type, a populated renal disease field or systematic comorbidity inventory, a complete concomitant medication history, or adjudicated reasons for treatment changes. Accordingly, HIV type 1 (HIV-1) infection and virological suppression were not assumed, and these characteristics could not be used for adjustment. Recorded PI exposure does not represent total polypharmacy. Treatment discontinuation indicators alone cannot distinguish adverse effects, virological failure, death, loss to follow-up or other reasons for stopping treatment.

Binary variables were harmonised as 0/1; missing entries remained unknown and were excluded from the observed denominator for that variable and from complete-case association analyses requiring it, without excluding the patient from the overall cohort. Prediction analyses instead imputed missing predictors using the relevant training fold only; missing outcomes were not imputed (Section 2.5). One stop TAF code of 0.93 and two death codes of 2 were invalid for binary fields and set to missing (Supplementary Table S1). Source PCr ratios were multiplied by 1000 for reporting in mg/g, retaining the original scale in the shared source workbook. Biochemical values outside the original inclusive plausibility ranges were set to missing: creatinine 0.2–15 mg/dL, calcium 4–15 mg/dL, phosphorus 0.5–10 mg/dL and vitamin D 0–200 ng/mL. The numbers excluded were one pre-TDF and one TDF-phase creatinine value, five calcium values in each phase, two TDF-phase and one TAF-phase phosphorus values, and no vitamin D values. No TAF-phase creatinine value was excluded. These broad data-screening bounds are not clinical reference intervals; detailed denominators and counts are provided in Supplementary Table S2.

For modelling, recorded TDF exposure accrued by switch was calculated from the selected episode start to the earlier of its stop date and the TAF start date. Eight stop dates later than the TAF start were capped at switch to prevent use of future exposure; two episodes starting after the recorded TAF start had unresolvable duration and were set to missing. No duration was inferred when the required dates were missing. This variable was expressed in years and does not quantify all previous TDF courses. The original source field for exposure longer than 96 weeks was retained only as a descriptive indicator. PI exposure was also set to missing for the two episodes starting after switch because those episode records could not establish pre-switch PI exposure.

### 2.3. Laboratory Procedures

All laboratory measurements were obtained as part of routine HIV outpatient monitoring; no study-specific sampling or additional laboratory testing was performed. Serum creatinine, total calcium, and inorganic phosphorus were measured on Roche cobas c analysers using routine photometric or colourimetric methods. According to the manufacturer’s method sheets used for the local assays, creatinine was measured with the Roche Creatinine Jaffé Gen.2 assay, calcium with the Roche Calcium Gen.2 assay, and phosphorus with the Roche Phosphate (Inorganic) ver.2 assay. Total 25-hydroxyvitamin D was measured on Roche cobas e immunoassay analysers using the Elecsys Vitamin D total III assay, an electrochemiluminescence binding immunoassay based on a competition principle.

The creatinine assay was traceable to isotope dilution mass spectrometry, the calcium assay was standardised against Standard Reference Material (SRM) 956c Level 2 reference material; the phosphorus assay was standardised against the New England Reagent Laboratory (NERL) primary reference material and, for the United States of America, National Institute of Standards and Technology (NIST)-traceable primary reference material; and the vitamin D assay was standardised using internal standards traceable to the isotope dilution liquid chromatography–tandem mass spectrometry (ID-LC-MS/MS) reference measurement procedure. For descriptive interpretation, adult laboratory reference intervals were those used by the local laboratory: serum creatinine 0.67–1.17 mg/dL for males and 0.51–0.95 mg/dL for females, inorganic phosphorus 2.5–4.5 mg/dL, total calcium 8.6–10.2 mg/dL for individuals aged 12–90 years and 8.2–9.6 mg/dL for those aged 90–100 years. Vitamin D status was categorised as normal when total 25-hydroxyvitamin D was greater than 30 ng/mL, insufficient when 10–30 ng/mL, and deficient when lower than 10 ng/mL.

eGFR was recalculated using the Chronic Kidney Disease Epidemiology Collaboration (CKD-EPI) 2021 creatinine equation [22]. Because specimen collection dates were unavailable, age at switch was used at both nominal laboratory phases; eGFR was missing when this age or another equation input was unavailable. These are approximate creatinine-based estimates rather than estimates using a verified age on each sampling date. The revised calculation avoids using age at the much later data extraction date in models intended to describe the switch period.

### 2.4. Definitions

Laboratory columns were labelled “TDF 96 weeks” and “TAF 48 weeks” in the source spreadsheet. These are nominal phase labels: specimen dates, allowed measurement windows, baseline-to-switch intervals and actual post-switch assessment intervals were not available. Neither a median/range of measured follow-up times nor adjustment for time since switch could therefore be provided. TAF start dates were recorded for 254/364 patients; treatment dates were not used as substitutes for specimen dates. Figures consequently use TDF and post-switch TAF phase labels without asserting uniform 96- or 48-week observation times.

The primary binary endpoint was PCr reduction < 20%, coded 1 when 100 × (pre-switch PCr − post-switch PCr)/pre-switch PCr was below 20 and 0 when it was at least 20. A baseline value of zero or a missing value at either phase made the endpoint unevaluable. The 20% threshold was retained from the original analysis as an exploratory operational definition. It is not a validated threshold of biological recovery, clinically important change or change exceeding measurement variability. Continuous PCr change and cutoffs of 10%, 30% and 50% were examined to show dependence on dichotomisation.

Secondary descriptive endpoints were post-switch PCr ≥ 154 mg/g, creatinine increase ≥ 10%, and a composite of any of these findings or the primary endpoint. The PCr cutoff was the 75th percentile of all 288 available baseline PCr measurements in the 364-patient cohort, calculated using R quantile type 7; it is a cohort-relative threshold, not a clinical diagnostic cutoff. The percentage change in serum creatinine was calculated as:

$$\mathrm{Creatinine}\;\mathrm{change}\;(\%)=\frac{\mathrm{Post}\text{-}\mathrm{switch}\;\mathrm{creatinine}-\mathrm{Pre}\text{-}\mathrm{switch}\;\mathrm{creatinine}}{\mathrm{Pre}\text{-}\mathrm{switch}\;\mathrm{creatinine}}\times 100\;.$$

Positive values indicate an increase in serum creatinine; an increase ≥ 10% defined the creatinine-worsening endpoint. The composite was coded 1 if at least one observed component was positive, 0 only if all three components were observed and negative, and missing otherwise. A sensitivity analysis required all three components to be observed.

### 2.5. Statistical Analysis and Exploratory Prediction Modelling

Continuous variables were summarised by median and interquartile range (IQR), with means and standard deviations (SD) in the supporting tables; categorical variables were reported with observed denominators and missing counts. Patients with and without paired PCr were compared using descriptive standardised mean differences (SMDs) rather than significance tests of baseline balance. Follow-up and discontinuation indicators were described separately because their temporal ordering relative to laboratory assessment was not established.

Paired changes in PCr, creatinine, eGFR, calcium, phosphorus and vitamin D were tested using two-sided Wilcoxon signed-rank tests with the normal approximation, tie handling and continuity correction. Rank-based analysis was chosen for the skewed distributions and influential values. The signed-rank test assesses paired signed ranks; its interpretation as a test of a median shift requires symmetry of the difference distribution. An exact sign-test sensitivity analysis was therefore added for PCr. Benjamini–Hochberg (BH) adjustment was applied once to the six raw Wilcoxon *p*-values, forming a single biomarker comparison family [23]. The reporting thresholds were two-sided *p* < 0.05 for nominal exploratory tests and q < 0.05 for this six-test family. Regression and sensitivity analyses were interpreted through effect estimates and 95% confidence intervals (CIs), without confirmatory claims from repeated nominal tests.

The principal association model was ordinary least-squares regression of post-switch log(1 + PCr), adjusted for baseline log(1 + PCr), age at switch (years), female versus male sex, recorded TDF exposure accrued by switch (years), concomitant PI exposure and baseline eGFR. This six-variable set was defined for the revised analysis to limit complexity and retain interpretable clinical covariates; it was not selected by *p*-values. Complete cases were used, with heteroscedasticity-consistent type 3 (HC3) standard errors and t-based 95% CI. Residual patterns, leverage, Cook’s distance and the scaled-predictor condition number were inspected, with sensitivity analyses excluding the maximum baseline PCr or the reviewer-highlighted creatinine observation. A model of delta log(1 + PCr), with identical observations and predictors, was used to expose the algebraic link between baseline and change, not as independent corroboration [21]. Supporting ordinary logistic regressions used the same covariates for the binary < 10%, <20% and <30% reduction endpoints. Outcomes, coding, model purposes, sample sizes and diagnostics are detailed in the accompanying analytical documentation. An exploratory natural cubic spline replaced the linear TDF-duration term with a three-degrees-of-freedom basis, retaining the other covariates and the same complete cases. Pointwise HC3 intervals describe the fitted curve at reference covariate values; this assesses possible departure from linearity without establishing a causal duration effect [24].

Missing primary outcomes were not imputed as recovered or unrecovered. Baseline comparisons, a date-complete subgroup analysis and assumption-free extreme-case bounds described the consequences of missingness; none establishes that outcomes were missing at random. The unidentified record and erroneous birth date were retained in the clinical cohort with explicitly recorded data limitations. The exploratory heatmap included six standardised paired biomarker changes, with clustering used descriptively.

Exploratory prediction addressed whether baseline clinical covariates added discrimination beyond PCr alone. A single method, ridge-penalised logistic regression, was evaluated with three predictor sets: baseline log(1 + PCr) only; the other five covariates listed above without PCr; and all six covariates. Ridge shrinkage was chosen to constrain coefficient instability in the small sample [25]. The penalty was applied to standardised coefficients, leaving the intercept unpenalised. No post-switch marker, discontinuation status, dropout indicator, extraction date age or future TDF exposure entered the prediction models. Restricting inputs to pre-switch or switch-time information prevents direct use of the future outcome in prediction; it does not remove the endpoint’s mathematical coupling with baseline PCr [21,26].

All three sets used identical stratified five-fold outer cross-validation, with five-fold inner cross-validation confined to each outer training set. Inner folds selected the penalty by minimum log loss from 15 logarithmically spaced values from 0.0001 to 100. Missing predictors were median-imputed and variables centred/scaled using the relevant training fold only, including within inner tuning. Each participant received exactly one outer-test probability per predictor set. Area under the receiver operating characteristic curve (ROC AUC), Brier score, log loss, calibration intercept and slope were computed from these out-of-fold predictions. AUC intervals and paired AUC differences used 2000 patient-level bootstrap resamples of the fixed out-of-fold predictions; these intervals are conditional on the fitted cross-validation procedure and do not include full model-retraining uncertainty. Wilson intervals were calculated for the observed event proportions in calibration quintiles and are supplied in the aggregate calibration table in the accompanying analytical package [27]. The prediction reporting follows the relevant Transparent Reporting of a multivariable prediction model for Individual Prognosis or Diagnosis elements, updated for artificial intelligence (TRIPOD + AI) for outcome and predictor definition, missingness, resampling, performance and availability of code [26]. No clinical action threshold or net clinical benefit was established.

Additional prediction diagnostics used the same held-out observations. Decision curves were constructed by calculating net benefit across hypothetical probability thresholds ranging from 0.05 to 0.80:

$$\mathrm{Net}\;\mathrm{benefit}=\frac{TP}{n}-\frac{FP}{n}\times \frac{t}{1-t}\;.$$

Here, *TP* and *FP* denote the numbers of true-positive and false-positive classifications, respectively; *n* is the number of participants; and *t* is the probability threshold. Classifying all participants as positive or all as negative provided the reference strategies. These thresholds were hypothetical and did not represent validated clinical indications [28]. Because the endpoint and associated clinical action are not validated, these are exploratory weighted-classification summaries and do not demonstrate clinical benefit. Permutation importance measures the decrease in pooled held-out AUC after shuffling one predictor within each outer test fold, retaining the fitted training transformations and selected penalty. Thirty repetitions describe permutation variability; negative decreases are retained. Importance does not establish causal effects and remains sensitive to the endpoint’s dependence on baseline PCr.

All statistical analyses were personally performed, checked and interpreted by Tommaso D’Anna (T.D.) using R version 4.6.1 (64-bit Universal C Runtime, UCRT) on Windows 11 (build 26200). The analytical and graphical workflow used stats, splines, graphics, grDevices, grid and utils, all version 4.6.1. The base, methods, datasets and compiler packages (also version 4.6.1) formed part of the R execution environment. Source workbook preprocessing used Python version 3.12.14 and openpyxl version 3.1.5; additional numerical checks used Python. Full software and package details are provided in the accompanying analytical documentation.

OpenAI Codex (GPT-6 Astra) provided ancillary assistance with code refinement, troubleshooting and language editing. T.D. checked the analytical code and numerical results and retains responsibility for the analytical decisions and interpretation. Verification covered source-data reconstruction, endpoint counts, multiplicity adjustment, regression coefficient identities, training/test separation and numerical optimisation. Separate Python calculations checked AUC, Brier scores, the exact PCr sign test and follow-up counts. Supplementary Methods, de-identified study data, executable scripts, session information, random seeds, reproduction commands, additional results and numerical validation outputs are supplied in Supplementary Material S2.

## 3. Results

The reconstructed cohort comprised 364 patients from 391 source treatment records. Switch dates were available for 254 (69.8%), with mean age at switch 47.9 years (SD 10.3) and median 48.5 years; age recorded at extraction was not used as a baseline predictor. Sex was recorded for 363 patients, of whom 262 were male and 101 female. HBV coinfection was recorded in 43/348 (12.4%) and HCV in 57/357 (16.0%); missing entries were not counted as absence. Paired PCr was available for 159/364 (43.7%), leaving 205 without the primary outcome. The paired and unpaired groups were a similar observed age at switch, sex distribution and baseline PCr; baseline calcium differed modestly (SMD 0.21). Variable-specific missingness was substantial and differed across groups (Table 1; Supplementary Table S3). Dropout was recorded in 18/159 (11.3%) with paired PCr and 62/205 (30.2%) without it; this was a retrospective status comparison, not a baseline predictor or an adjudicated explanation for missingness.

In 159 patients with paired PCr, median values were 102.0 mg/g during TDF and 88.0 mg/g after switch. Median within-person change was −4.0 mg/g (IQR −41.5 to 11.5), whereas the mean change was −125.7 mg/g, reflecting a markedly skewed distribution. The Wilcoxon *p*-value was 0.00655 and the six-biomarker BH-adjusted q-value 0.00983 (Table 2A). PCr decreased in 85 patients, increased in 64 and was unchanged in 10; an exact two-sided sign test excluding ties gave *p* = 0.1010 (Supplementary Table S4). Thus, the signed-rank finding does not establish significantly more decreases than increases or, without the symmetry assumption, a significant median improvement. The <20% reduction endpoint occurred in 99/159 (62.3%); the corresponding frequencies at 10%, 30% and 50% were 87/159 (54.7%), 112/159 (70.4%) and 137/159 (86.2%), respectively (Supplementary Table S5). These percentages describe the chosen thresholds, not the prevalence of persistent tubular injury.

Post-switch PCr ≥ 154 mg/g occurred in 36/175 (20.6%); creatinine increase ≥10% in 79/226 (35.0%); and the any-positive composite in 162/188 classifiable patients (86.2%). Of 159 with all three components observed, 133 (83.6%) were composite-positive. The other 29 positive classifications had at least one component missing, and 176 patients remained unclassifiable (Table 2B; Supplementary Table S6). The creatinine median increased by 0.03 mg/dL and the eGFR median paired change was −2.24 mL/min/1.73 m^2^. These estimates describe laboratory change, with eGFR calculated using switch age as an approximation for both phases.

The baseline-adjusted post-switch PCr model included 155 complete cases. Baseline log(1 + PCr) had a coefficient of 0.534 (HC3 95% CI 0.379–0.690; *p* = 2.64 × 10^−10^), indicating persistence of between-patient differences in proteinuria. With the same patients and covariates, its coefficient in the delta log(1 + PCr) model was −0.466 (95% CI −0.621 to −0.310), exactly one unit lower. The other coefficients and residuals were unchanged by this re-expression of the outcome; these models are algebraically related and do not provide independent evidence of biological recovery. PI exposure was not associated at the nominal 0.05 level in the revised model (coefficient −0.158; 95% CI −0.373 to 0.057). The supporting logistic analysis also associated higher baseline log(1 + PCr) with lower odds of the <20% reduction endpoint (odds ratio [OR] 0.284; 95% CI 0.160–0.506), with the same endpoint-coupling limitation (Table 3).

Figure 1 displays paired PCr trajectories, the coefficient plot, duration-stratified summaries and a spline curve. The log(1 + PCr) scale makes the dense low-PCr range visible while retaining the extreme observations. The coefficient panel displays the change-model estimates with HC3 intervals; its baseline association includes mathematical coupling. The duration group panel shows observed medians and IQRs among the same patients at both phases: 41 had exposure ≤96 weeks, 116 had exposure >96 weeks and two lacked a valid accrued duration. Groups refer to the selected TDF episode accrued by switch and do not represent verified laboratory follow-up intervals. In the exploratory spline model (n = 155), HC3 Wald tests gave *p* = 0.437 for joint nonlinearity and *p* = 0.629 for the overall duration association. These checks do not establish a duration effect or biological recovery (Supplementary Tables S7 and S8).

The heatmap was reconstructed from six standardised paired biomarker changes in 110 complete profiles (Figure 2). It is a descriptive display of within-patient variation, not evidence for discrete phenotypes. Each anonymous row represents a complete biomarker-change profile. The observation highlighted by the reviewer had creatinine 1.03 mg/dL during TDF and 10.8 mg/dL after switch (change +9.77 mg/dL). The source workbook confirms that this is the recorded value, but cannot establish its clinical explanation or exclude a transcription error. It lies within the retained broad plausibility bounds and was not automatically deleted. Excluding it reduced the mean creatinine change from 0.095 to 0.052 mg/dL, while the median remained 0.03 mg/dL; the signed-rank sensitivity *p*-value was 0.00000589 (Supplementary Table S9). No acute kidney injury diagnosis was inferred from this isolated record.

Influence analyses retained the positive baseline-PCr association with post-switch PCr: its coefficient was 0.564 (95% CI 0.397–0.730) after removing the maximum baseline-PCr observation and 0.535 (0.379–0.691) after removing the highlighted creatinine observation. The complete case model had 10 observations above the descriptive Cook’s distance threshold of 4/n; these flags were used to inspect influence rather than to impose additional exclusions. All 159 paired-PCr cases belonged to the subgroup with recorded switch dates, so restricting to that subgroup left the primary 99/159 frequency unchanged. Using its baseline-PCr quartile (151.25 rather than 154 mg/g) classified 37 rather than 36 of the 175 post-switch measurements as high. These checks do not resolve selection arising from the 205 missing primary outcomes. Without assumptions about those outcomes, the logical full-cohort frequency could range from 99/364 (27.2%) to 304/364 (83.5%); these are extreme-case bounds, not an estimated cohort rate.

Nested cross-validation gave an AUC of 0.709 (conditional bootstrap 95% CI 0.622–0.792) for PCr alone, 0.475 (0.382–0.568) without PCr, and 0.701 (0.609–0.781) for the full six-variable set. Brier scores were 0.203, 0.235 and 0.205, respectively (Table 4). The full-minus-PCr-only AUC difference was −0.009 (95% CI −0.056 to 0.039), providing no evidence of improved discrimination from the additional variables in these data. The no-PCr model produced almost constant probabilities near the observed event frequency and did not provide useful discrimination.

Calibration plots use the same held-out predictions as the ROC analysis (Figure 3 and Figure 4). For PCr alone, the calibration intercept was approximately 0 and slope 0.995; for the full set they were 0.004 and 1.142. These summaries have substantial sampling uncertainty, particularly within small risk strata. Calibration slope was unstable for the nearly constant no-PCr predictions and is not evidence of a usable model. Predicted risks overlapped between observed outcome groups: the median PCr-only probability was 0.607 in those with ≥20% reduction and 0.683 in those with <20% reduction. This overlap limits individual classification despite the moderate AUC. No monitoring benefit, clinical action threshold or externally validated prediction rule was demonstrated.

The decision curves and permutation importance profiles are shown in Figure 3 alongside calibration. The decision curve thresholds represent hypothetical classification preferences, not validated indications for changing treatment or monitoring. Permuting baseline PCr within held-out folds reduced pooled AUC by a mean of 0.204 (permutation SD 0.045) in the PCr-only set and 0.195 (SD 0.041) in the full set. Negative decreases for some other variables were retained. These are model reliance diagnostics, not additional evidence of a biological effect: baseline PCr also enters the outcome definition. Detailed estimates and their calculation are supplied in Supplementary Tables S10 and S11.

## 4. Discussion

In this single-centre retrospective cohort, proteinuria trajectories after TDF-to-TAF switching were heterogeneous. The established renal safety evidence for TAF derives from comparative trials and other cohorts [7,8,9,29,30,31]; our uncontrolled before–after analysis cannot independently establish a treatment benefit. Published TDF withdrawal and switch cohorts also use different renal outcomes, which limits direct comparison of their recovery frequencies with our operational <20% PCr reduction endpoint [11,12,13,14,29,30].

Recent observational studies extend the long-term context. Gilbert et al. reported a median eGFR decrease over 3.6 years after TDF-to-TAF switching [20]. In a Japanese cohort followed for 288 weeks, urinary β2-microglobulin decreased despite later eGFR decline [19]. Chen et al. compared 2422 TAF-treated participants with 252 non-tenofovir controls: long-term eGFR decline did not differ significantly, total proteinuria remained stable, and urinary β2-microglobulin initially decreased [18]. These studies reinforce the need to distinguish total proteinuria, tubular markers and creatinine-based filtration estimates. They do not establish the cause of PCr changes in our cohort.

Older age, pre-existing renal impairment, prolonged TDF exposure and concomitant treatment have been associated with renal outcomes in previous cohorts [6,14,30]. Our available covariates describe only part of this clinical context; an observed association with PI exposure would not establish that PI use protects against proteinuria or that stopping TDF reverses an injury caused by the PI-containing regimen. Unmeasured comorbidity, HIV disease activity, medication changes and treatment selection patterns remain plausible contributors.

Cobicistat and dolutegravir can increase serum creatinine through inhibition of tubular secretion without a corresponding reduction in measured glomerular filtration rate (GFR) [32,33]. In the absence of adequate longitudinal drug exposure information, cystatin C or measured GFR, their contribution to the creatinine/eGFR changes in this cohort cannot be determined.

Total PCr remains a practical measure of proteinuria, but the present findings should not be interpreted as validation of PCr as a specific marker of tubular injury. Neither albumin-to-total-protein fractionation nor low-molecular-weight tubular proteins were available. The association between baseline PCr and a percentage-change outcome is partly built into the endpoint: baseline appears both as a predictor and in the calculation of change. Higher starting values also provide more scope for reduction, and measurement variability can generate regression to the mean. A person with a low baseline PCr may fail to achieve a 20% decrease while retaining a low post-switch value; conversely, a substantial proportional decrease may leave clinically important proteinuria. Continuous post-switch PCr and absolute renal measurements must therefore be interpreted alongside the relative-change classification [15,16,17,21].

Assessment of suspected treatment-related renal harm requires separating a biochemical finding, a clinically characterised injury and attribution of that injury to a particular exposure. In pharmacovigilance, temporal compatibility, competing diseases or medicines, the course after withdrawal and the quality of the underlying documentation inform case causality assessment; even structured assessment does not prove an individual drug event connection [34]. These elements were incompletely recorded here, and no formal case-level causality assessment was performed. Similarly, a single post-switch PCr value or failure to cross a percentage threshold cannot establish permanence, irreversibility or the extent of functional impairment. Interpretation therefore depends on documented serial measurements and clinical context rather than the relative-change category alone.

TDF exposure and TDF-to-TAF switching have also been studied in relation to bone safety [2,7,8,9]. The available densitometry in this cohort was too incomplete to support an analysis of BMD recovery or fracture outcomes. Changes in serum calcium, phosphorus or vitamin D do not establish skeletal recovery, and these exploratory laboratory comparisons should not be interpreted as evidence of an effect on fractures.

The exploratory prediction analysis addresses a different question from testing a regression coefficient: it evaluates how well-defined predictor sets distinguish the operational endpoint in held-out observations. Comparing sets with and without baseline PCr helps show how much apparent discrimination depends on a variable that also defines the outcome [21]. Even a well-discriminating model of this endpoint would not demonstrate prediction of irreversible injury. Overlap in predicted probabilities between outcome groups, calibration uncertainty and the absence of external validation limit interpretation, and no clinical decision rule or benefit from model-guided care was established [26,27].

Several limitations constrain interpretation. The retrospective before–after design lacked a concurrent group remaining on TDF. Changes in accompanying ART, other medication, HIV disease activity, intercurrent illness, renal disease and usual care may have contributed to the observed trajectories. The analyses therefore describe changes after switching and do not estimate a causal effect of TAF or TDF withdrawal.

More than half of the cohort lacked paired PCr values. The direct comparison of observed baseline characteristics helps describe selection into the analytical subgroup, but cannot exclude selection by unmeasured characteristics or outcome-dependent monitoring. The rate of a PCr reduction below 20% applies to patients with evaluable pairs and cannot be extrapolated to the entire clinic cohort. Sensitivity analyses depend on their own assumptions and cannot recover unobserved outcomes without additional information.

Laboratory fields were labelled by nominal treatment phases, without specimen collection dates or permitted measurement windows. The baseline-to-switch interval and actual post-switch follow-up distribution could not be calculated; no time-since-switch adjustment was possible. These omissions prevent the results from being interpreted as a uniform 48-week trajectory. CD4 count, viral load, HIV type, detailed comorbidities and concomitant medications were unavailable. Reasons for TAF discontinuation were not adjudicated, so treatment tolerability and reasons for missing follow-up could not be evaluated reliably.

The primary relative-change threshold was not clinically validated and total PCr is not tubular-specific. Mathematical coupling, regression to the mean and influential observations affect its interpretation. Plausibility screening cannot adjudicate a clinically possible extreme laboratory result as genuine or erroneous. Prediction modelling was exploratory and internally evaluated only; the sample was small, missing predictors required assumptions, and neither clinical benefit nor transportability was demonstrated.

The selected TDF episode ended on the recorded TAF start date in 134 of the 254 date-complete records, preceded it in 112 and extended beyond it in eight. Consequently, the available treatment records do not establish immediate substitution or uninterrupted exposure for every participant. Capping exposure at switch prevents use of future duration but cannot resolve inconsistent treatment chronology. The completeness-based row-selection rule and the unavailable corrected birth date are further source-data limitations.

## 5. Conclusions

In this single-centre cohort, PCr changes following TDF-to-TAF switching were heterogeneous. A reduction below the operational 20% threshold was common among patients with paired measurements. Unavailable specimen timing and clinical corroboration preclude classifying these observations as persistent or irreversible drug-related injury in individual patients. The findings also emphasise the value of a documented clinical chronology and explicit consideration of alternative causes when assessing suspected treatment-related renal harm in individual patients. Baseline PCr contributed information about this relative-change endpoint while also being mathematically coupled to its definition. The findings support further study with measured follow-up intervals, tubular-specific biomarkers, better clinical covariate ascertainment and external validation; they do not establish a causal treatment effect or a prediction rule for clinical use.

## Figures and Tables

**Figure 1:**
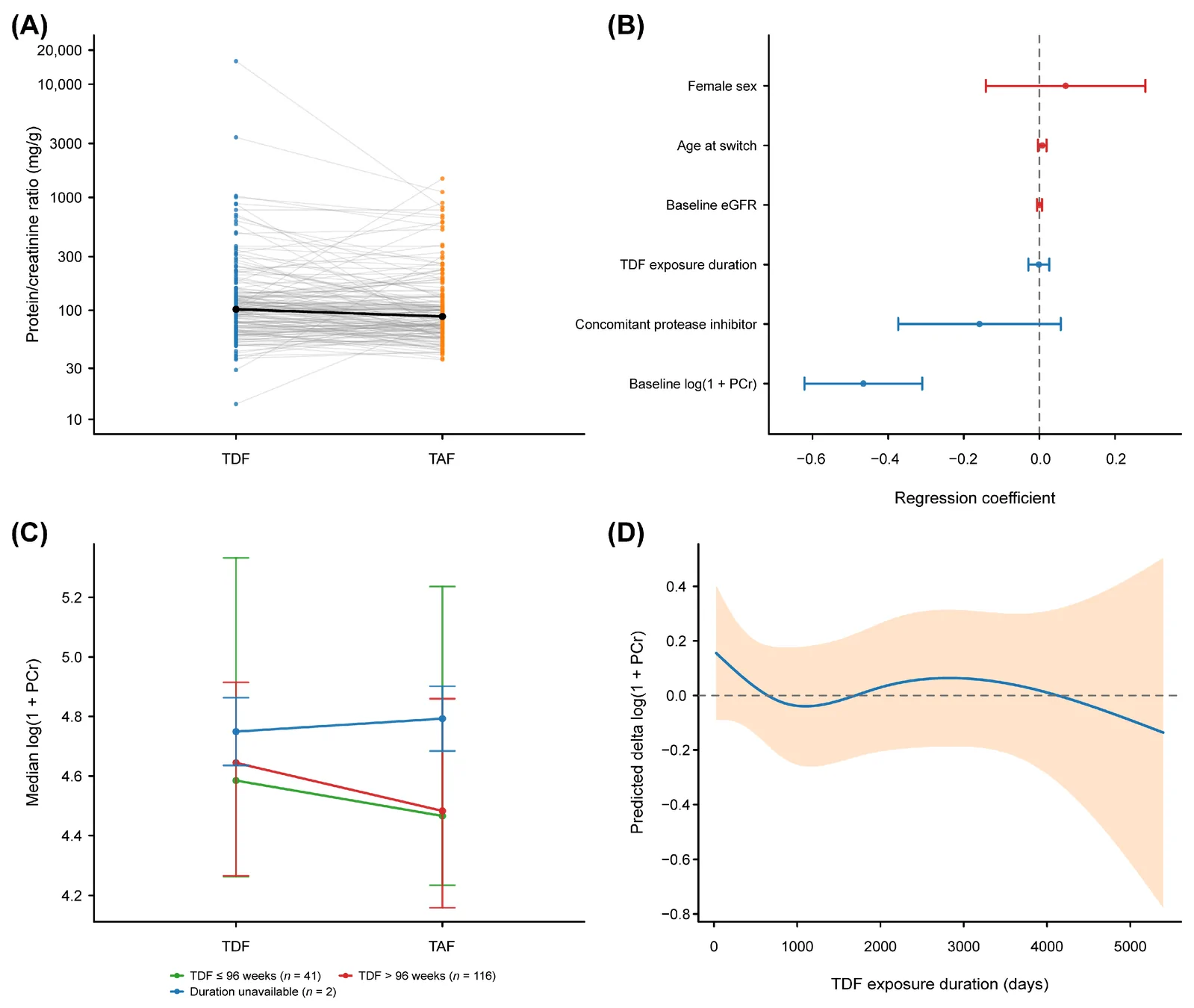
Paired proteinuria changes and regression analyses after switching from TDF to TAF. (**A**) Paired urinary protein-to-creatinine ratio (PCr) in 159 patients. Grey lines connect individual measurements; blue and orange points denote TDF and TAF phases; and black points/line denote medians. The log(1 + PCr) axis has mg/g tick labels; phase labels do not imply verified specimen intervals. (**B**) Change-model coefficients and HC3 95% confidence intervals in 155 complete cases, with negative coefficients blue and positive coefficients red. Age and accrued TDF exposure are in years. The dashed line marks zero. Baseline PCr also enters the change formula, so its coefficient includes mathematical coupling. (**C**) Observed medians and interquartile ranges in the same paired patients, grouped by TDF exposure accrued by switch: ≤96 weeks (n = 41), >96 weeks (n = 116), or unavailable (n = 2). These are descriptive summaries, not adjusted estimates or confidence intervals. (**D**) Exploratory natural cubic spline with three degrees of freedom and the remaining five covariates (n = 155). The blue curve and orange band show the fitted conditional mean and pointwise HC3 95% intervals at median continuous covariates, male sex and no PI exposure. The dashed line marks zero change; the curve does not estimate a causal duration effect. Abbreviations: TDF, tenofovir disoproxil fumarate; TAF, tenofovir alafenamide; eGFR, estimated glomerular filtration rate; PI, protease inhibitor; HC3, heteroscedasticity-consistent covariance estimator type 3; CI, confidence interval.

**Figure 2:**
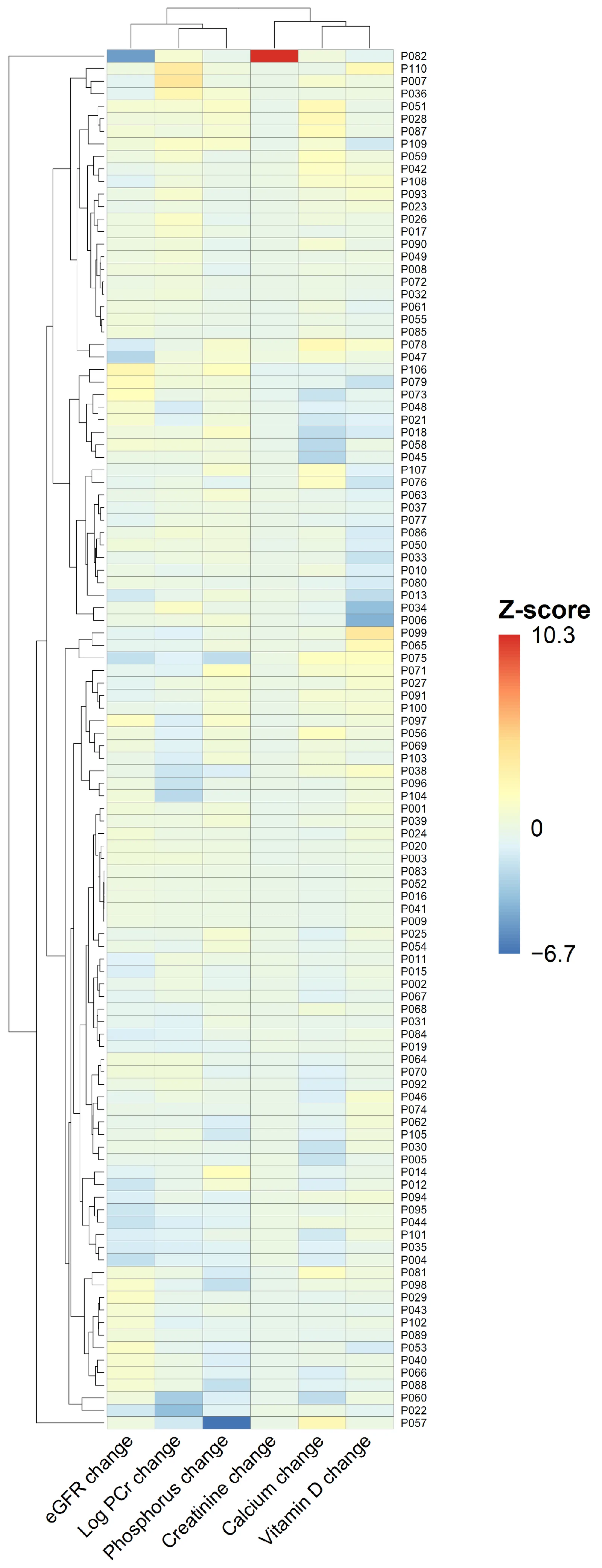
Standardised paired biomarker changes in 110 patients with complete profiles. Anonymous rows show TAF-minus-TDF changes; PCr uses log(1 + PCr). Each variable was centred and scaled using these 110 profiles. Rows and columns use complete-linkage Euclidean clustering; upper dendrogram heights are compressed for display. Colours retain the full observed Z-score range, approximately −6.7 to 10.3, without clipping. Red and blue represent higher and lower changes relative to each variable’s mean; they do not uniformly indicate improvement or deterioration. The first row (P082) includes recorded creatinine 1.03 to 10.8 mg/dL; its clinical cause is undetermined. Abbreviations: PCr, urinary protein-to-creatinine ratio; TDF, tenofovir disoproxil fumarate; TAF, tenofovir alafenamide; eGFR, estimated glomerular filtration rate.

**Figure 3:**
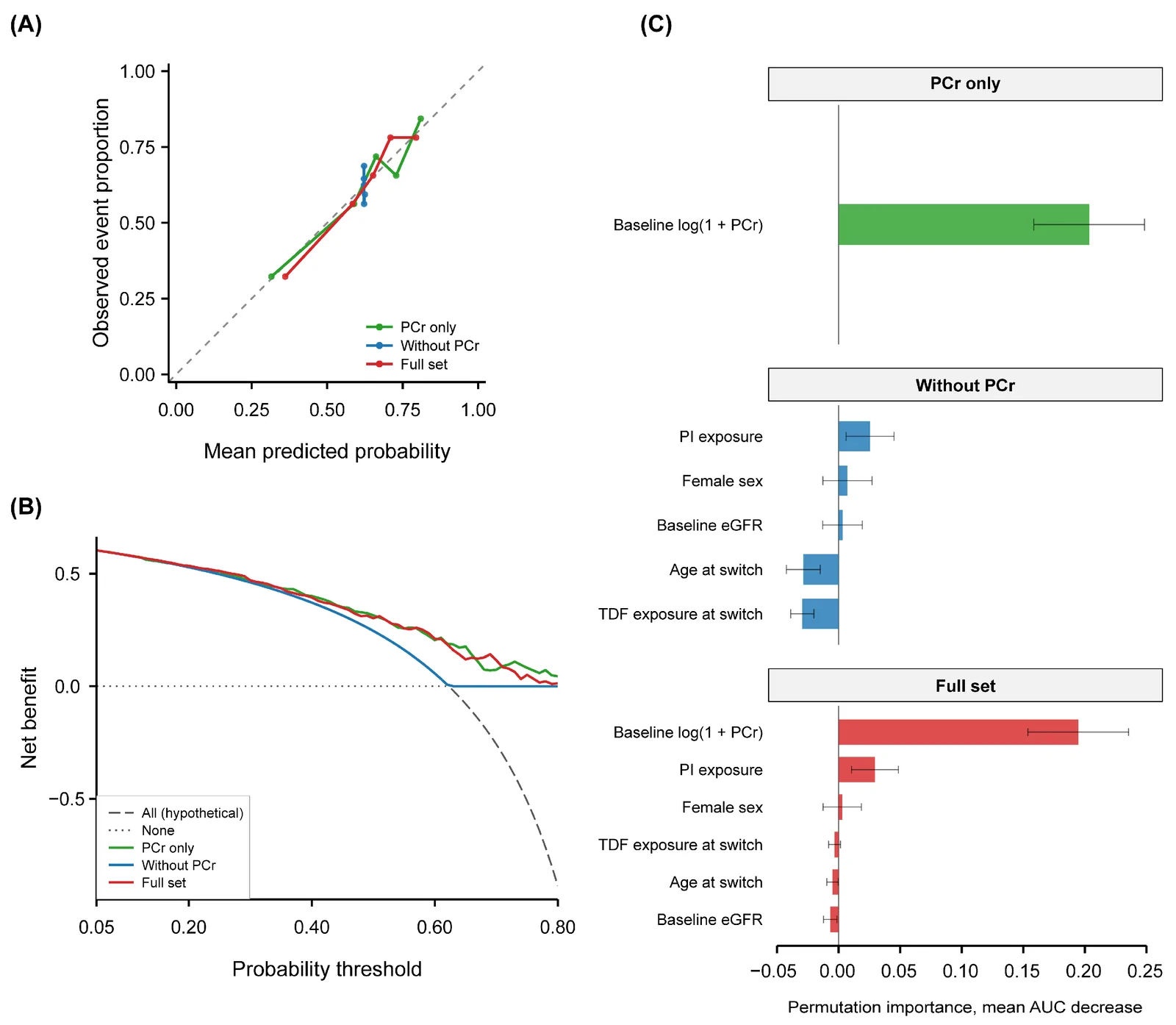
Calibration, hypothetical threshold analysis and held-out predictor importance for the three ridge-logistic predictor sets. All panels use the same 159 patients and the final outer-fold predictions from nested cross-validation. Green denotes baseline log(1 + PCr) alone, blue the other five covariates without PCr, and red the full predictor set. (**A**) Mean predicted probability and observed proportion with a PCr reduction < 20% in five approximately equal-sized prediction groups (31 or 32 patients per group). Points are connected within predictor sets; the dashed diagonal denotes agreement. No confidence intervals are plotted; the corresponding Wilson intervals are supplied in the supplementary aggregate calibration table. The nearly constant no-PCr predictions produce overlapping groups and do not demonstrate useful stratification. (**B**) Exploratory net-benefit curves over hypothetical probability thresholds of 0.05–0.80, calculated as TP/n − (FP/n) × threshold/(1 − threshold). “All” and “None” are hypothetical reference classifications. No clinical action or decision threshold was validated; these curves do not establish benefit from model-guided care. (**C**) Mean decrease in pooled held-out AUC after permuting each predictor within outer test folds while retaining the corresponding fitted model. Bars show means across 30 permutations; horizontal error bars show permutation standard deviations, not confidence intervals. Negative values are retained. Importance describes model sensitivity to a predictor, not causation, and baseline PCr also forms part of the endpoint definition. Abbreviations: PCr, urinary protein-to-creatinine ratio; TDF, tenofovir disoproxil fumarate; eGFR, estimated glomerular filtration rate; PI, protease inhibitor; AUC, area under the curve; TP, true-positive classifications; FP, false-positive classifications.

**Figure 4:**
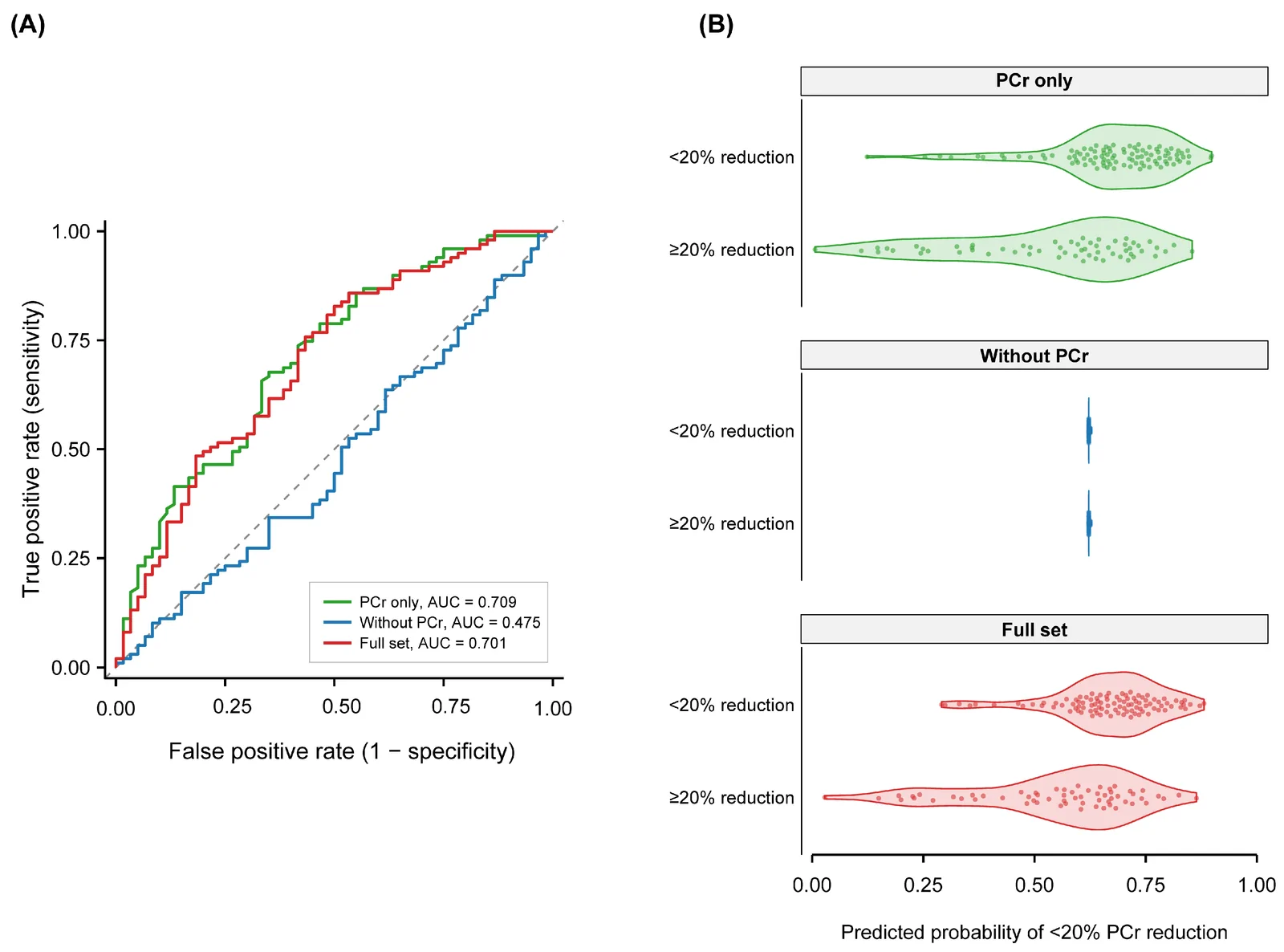
Cross-validated ROC curves and individual predicted probability distributions for the three ridge-logistic predictor sets. (**A**) Receiver-operating-characteristic curves from the final outer-fold predictions, with AUC values in the legend; the dashed diagonal denotes chance discrimination. AUC confidence intervals are reported in Table 4. (**B**) Horizontal violin plots show predicted probabilities by observed PCr response: reduction ≥ 20% (60 patients) or < 20% (99 patients). Each violin is a Gaussian kernel density estimate using the normal reference bandwidth selected by R’s density function (bw = “nrd0”), trimmed to the observed probability range and scaled to equal maximum width. Each point represents one patient’s outer-held-out prediction for that predictor set; deterministic displacement along the category axis improves visibility without changing the probability coordinate. The same 159 patients appear in each of the three set-specific displays, with one prediction per patient per set and no repeated-fold predictions counted as additional patients. The no-PCr distributions are narrow because predictions lie near the event prevalence. Overlap between response groups limits individual classification, and no clinical intervention threshold is established. Colours and predictor set labels match Figure 3. Abbreviations: PCr, urinary protein-to-creatinine ratio; ROC, receiver operating characteristic; AUC, area under the curve.

**Table 1:** Observed baseline characteristics in the full cohort and according to availability of paired PCr.

Characteristic	Overall (N = 364)	Paired PCr (n = 159)	No Paired PCr (n = 205)	SMD
Age at switch, years	48.46 [41.39, 54.55]; n = 254	48.48 [41.40, 55.05]; n = 159	48.37 [41.44, 53.82]; n = 95	0.12
Time from first HIV contact to switch, years	7.49 [4.20, 14.84]; n = 248	7.60 [4.29, 14.97]; n = 158	7.04 [3.89, 14.36]; n = 90	−0.04
Recorded TDF exposure accrued by switch, years	3.51 [1.78, 6.06]; n = 252	3.75 [1.74, 6.23]; n = 157	3.27 [1.95, 5.51]; n = 95	−0.01
Baseline creatinine, mg/dL	0.88 [0.76, 1.00]; n = 340	0.87 [0.78, 0.99]; n = 159	0.88 [0.76, 1.01]; n = 181	−0.04
Baseline eGFR, mL/min/1.73 m^2^	100.76 [89.10, 108.85]; n = 238	100.71 [89.65, 108.43]; n = 159	100.81 [88.93, 108.95]; n = 79	−0.05
Baseline PCr, mg/g	102.00 [70.00, 154.00]; n = 288	102.00 [70.00, 146.50]; n = 159	100.00 [71.00, 156.00]; n = 129	0.06
Baseline calcium, mg/dL	9.34 [9.04, 9.60]; n = 324	9.39 [9.09, 9.65]; n = 154	9.30 [9.00, 9.59]; n = 170	0.21
Baseline phosphorus, mg/dL	3.10 [2.70, 3.50]; n = 328	3.05 [2.70, 3.40]; n = 157	3.10 [2.73, 3.50]; n = 171	−0.04
Baseline vitamin D, ng/mL	24.45 [19.00, 31.77]; n = 216	25.00 [19.76, 33.14]; n = 117	24.23 [18.80, 31.51]; n = 99	0.07
Female sex	101/363 (27.8%)	44/159 (27.7%)	57/204 (27.9%)	−0.01
HBV coinfection	43/348 (12.4%)	19/154 (12.3%)	24/194 (12.4%)	0.00
HCV coinfection	57/357 (16.0%)	22/156 (14.1%)	35/201 (17.4%)	−0.09
PI exposure during TDF	234/358 (65.4%)	107/155 (69.0%)	127/203 (62.6%)	0.14
Recorded TDF exposure > 96 weeks	260/361 (72.0%)	118/158 (74.7%)	142/203 (70.0%)	0.11

Continuous variables are median [IQR], with the observed n shown in each cell; categorical variables are n/N (%). SMD is paired minus unpaired, based on observed values. No SMD establishes absence of selection bias. Age and eGFR refer to the switch period; the recorded TDF episode duration is capped at switch and does not represent lifetime exposure. Full missingness and separate follow-up status summaries are in Supplementary Table S3. Abbreviations: PCr, urinary protein-to-creatinine ratio; TDF, tenofovir disoproxil fumarate; HIV, human immunodeficiency virus; HBV, hepatitis B virus; HCV, hepatitis C virus; eGFR, estimated glomerular filtration rate; PI, protease inhibitor; IQR, interquartile range; SMD, standardised mean difference.

**Table 2:** Paired laboratory changes and descriptive endpoint frequencies. (**A**) Paired biomarker comparisons. (**B**) Descriptive binary endpoints.

(**A**)						
**Biomarker**	**Pairs**	**TDF Median [IQR]**	**TAF Median [IQR]**	**Paired Change [IQR]**	**Wilcoxon *p***	**BH q**
Creatinine	226	0.86 [0.76, 0.99]	0.90 [0.79, 1.02]	0.03 [−0.04, 0.13]	3.88 × 10^−6^	2.33 × 10^−5^
eGFR	226	101.05 [90.02, 108.85]	97.96 [81.97, 107.44]	−2.24 [−10.72, 2.93]	8.77 × 10^−6^	2.63 × 10^−5^
PCr	159	102.00 [70.00, 146.50]	88.00 [63.50, 132.50]	−4.00 [−41.50, 11.50]	0.0066	0.0098
Calcium	201	9.35 [9.04, 9.60]	9.48 [9.21, 9.76]	0.06 [−0.14, 0.36]	3.99 × 10^−4^	7.99 × 10^−4^
Phosphorus	209	3.08 [2.70, 3.42]	2.99 [2.62, 3.36]	−0.07 [−0.41, 0.34]	0.1993	0.2392
Vitamin D	154	25.00 [19.35, 32.70]	24.00 [18.73, 31.20]	0.00 [−4.00, 3.39]	0.3481	0.3481
(**B**)						
**Endpoint**			**Evaluable n**		**Events n (%)**	
PCr reduction < 20%			159		99 (62.3%)	
Post-switch PCr ≥ 154 mg/g			175		36 (20.6%)	
Creatinine increase ≥ 10%			226		79 (35.0%)	
Composite, any observed positive			188		162 (86.2%)	
Composite, all components observed			159		133 (83.6%)	

Phase values and paired changes are median [IQR]. PCr is mg/g; creatinine, calcium and phosphorus are mg/dL; vitamin D is ng/mL; eGFR is mL/min/1.73 m^2^. Each biomarker uses its own complete pairs. The q column is Benjamini–Hochberg adjustment of exactly the six Wilcoxon *p*-values in Table 2A. The composite is positive if any observed component is positive and negative only if all three are observed and negative; otherwise, it is missing. These relative or cohort-derived thresholds are not validated clinical recovery criteria. Abbreviations: PCr, urinary protein-to-creatinine ratio; TDF, tenofovir disoproxil fumarate; TAF, tenofovir alafenamide; eGFR, estimated glomerular filtration rate; IQR, interquartile range; BH, Benjamini–Hochberg.

**Table 3:** Baseline-adjusted association models using the six-variable revised covariate set. (**A**) Linear regression of post-switch log(1 + PCr), n = 155. (**B**) Supporting logistic regression of PCr reduction < 20%, n = 155.

(**A**)			
**Covariate**	**Coefficient**	**HC3 95% CI**	***p***
Baseline log(1 + PCr)	0.5343	0.3786 to 0.6900	2.64 × 10^−10^
Age at switch, years	0.0076	−0.0037 to 0.0189	0.1851
Female sex	0.0694	−0.1416 to 0.2804	0.5167
TDF exposure by switch, years	−0.0015	−0.0292 to 0.0262	0.9166
PI exposure	−0.1584	−0.3735 to 0.0566	0.1475
Baseline eGFR	0.0002	−0.0062 to 0.0067	0.9430
(**B**)			
**Covariate**	**OR**	**95% CI**	***p***
Baseline log(1 + PCr)	0.284	0.160 to 0.506	1.86 × 10^−5^
Age at switch, years	1.005	0.963 to 1.049	0.8030
Female sex	1.500	0.654 to 3.438	0.3384
TDF exposure by switch, years	1.029	0.912 to 1.160	0.6458
PI exposure	0.523	0.219 to 1.250	0.1449
Baseline eGFR	1.000	0.972 to 1.028	0.9768

Table 3A reports HC3 robust 95% CI. Table 3B reports ordinary logistic regression OR and Wald 95% CI; 97/155 complete cases had the endpoint. Female sex is relative to male sex, and PI exposure to no PI exposure. Continuous effects are per one year of age or recorded TDF exposure, one mL/min/1.73 m^2^ eGFR, or one log(1 + PCr) unit. In the corresponding change model, the baseline-PCr coefficient is exactly one unit lower than in the post-switch model. Supporting threshold regressions and diagnostics are reported in the Supplementary tables. Abbreviations: PCr, urinary protein-to-creatinine ratio; TDF, tenofovir disoproxil fumarate; eGFR, estimated glomerular filtration rate; PI, protease inhibitor; HC3, heteroscedasticity-consistent covariance estimator type 3; CI, confidence interval; OR, odds ratio.

**Table 4:** Nested cross-validated performance of one ridge-logistic method with three predictor sets.

Predictor Set	AUC	Conditional 95% CI	Brier Score	Log Loss
PCr only	0.709	0.622 to 0.792	0.203	0.593
Covariates without PCr	0.475	0.382 to 0.568	0.235	0.663
Full six-variable set	0.701	0.609 to 0.781	0.205	0.597

All sets used the same 159 patients, 99 events and outer folds, with one held-out probability per patient. AUC CI are from 2000 patient-level bootstrap resamples of fixed out-of-fold predictions and are conditional on that procedure. Lower Brier score and log loss indicate better probability prediction. The full set comprises baseline log(1 + PCr), switch age, sex, TDF exposure accrued by switch, PI exposure and baseline eGFR; the no-PCr set omits only baseline log(1 + PCr). Calibration and paired AUC differences are reported in the text and Supplementary tables. Abbreviations: PCr, urinary protein-to-creatinine ratio; TDF, tenofovir disoproxil fumarate; eGFR, estimated glomerular filtration rate; PI, protease inhibitor; AUC, area under the curve; CI, confidence interval.

## Data Availability

The de-identified data supporting this study are available in Supplementary Material S2. The package includes the 391-record source workbook, the 364-patient numeric reference and analytical dataset, held-out predictions, executable code, aggregate results, a data dictionary, software versions and reproduction instructions. Direct identifiers, birth dates, calendar dates and free-text regimen descriptions have been removed; random study keys and relative timing variables permit numerical reproduction without access to the identified clinical records. The original identified records and linkage file remain restricted and are not included in the Supplementary Materials.

## Supplementary Materials

The following supporting information can be downloaded at: https://www.mdpi.com/article/10.3390/diseases14100353/s1. Supplementary Material S1 contains the tables cited in the main text: Table S1. Invalid binary codes set to missing; Table S2. Inclusive biochemical plausibility bounds and exclusions; Table S3A. Cohort reconstruction and primary endpoint availability; Table S3B. Observed baseline comparison and available denominators; Table S3C. Retrospective follow-up indicators; Table S4. Exact PCr sign test sensitivity; Table S5. Sensitivity to the required PCr reduction; Table S6. Component and composite completeness; Table S7. Observed log(1+PCr) by accrued TDF exposure; Table S8. Exploratory natural-spline checks, n = 155; Table S9A. Influence of the highlighted creatinine observation; Table S9B. Baseline PCr coefficient in post-switch models; Table S10. Hypothetical decision curve summaries; Table S11. Held-out permutation importance; Table S12. Additional continuous baseline descriptions; Table S13. Linear model diagnostic summaries; Table S14. Baseline PCr association under alternative binary thresholds; Table S15. Nested cross-validation tuning; Table S16. Paired differences in nested cross-validated AUC; Table S17. Date-complete and endpoint definition checks; Table S18. Extraction time variables retained for description only; Table S19. Known and unknown follow-up status; Table S20. Logical bounds for the full-cohort primary endpoint. Supplementary Material S2 contains analytical methods, de-identified study data, executable code, additional results (Tables S12–S20), figure files and reproduction instructions. The analytical documentation is provided as Supplementary Informations within the archive.

## Abbreviations

The following abbreviations are used in this manuscript:

Abbreviation	Meaning
ART	Antiretroviral therapy
AUC	Area under the curve
BMD	Bone mineral density
CI	Confidence interval
CKD-EPI	Chronic Kidney Disease Epidemiology Collaboration
eGFR	Estimated glomerular filtration rate
HBV	Hepatitis B virus
HCV	Hepatitis C virus
HC3	Heteroscedasticity-consistent covariance estimator type 3
HIV	Human immunodeficiency virus
IQR	Interquartile range
OR	Odds ratio
PCr	Urinary protein-to-creatinine ratio
PI	Protease inhibitor
RBP	Retinol-binding protein
ROC	Receiver operating characteristic
SD	Standard deviation
SMD	Standardised mean difference
TAF	Tenofovir alafenamide
TDF	Tenofovir disoproxil fumarate
β2M	β2-microglobulin
