# Supplementary material for: Assessing Renal Outcomes After Switching from Tenofovir Disoproxil Fumarate to Tenofovir Alafenamide in People with HIV: Proteinuria Changes and Limits of Causal Attribution
Source: Diseases. 2026 Sep 24;14(10):353. doi: 10.3390/diseases14100353 (PMC13703067; doi:10.3390/diseases14100353)
Supplement: Supplementary file 1 [file diseases-14-00353-s001.zip › Supplementary Material S1.pdf]

Supplementary Material S1

This file contains Supplementary Tables S1–S11, numbered in order of first citation in the main manuscript. Lettered panels are retained within their respective tables. Analytical methods, de-identified data, executable code and additional results, including Tables S12–S20, are provided in Supplementary Material S2.

Table S1. Invalid binary codes set to missing.

Indicator	Invalid codes
HBV coinfection	0
HCV coinfection	0
Recorded renal disease	0
PI exposure during TDF	0
Recorded TDF >96-week indicator	0
Recorded TAF >48-week indicator	0
Stop-TAF indicator	1
Dropout indicator	0
Death indicator	2

Abbreviations: TDF, tenofovir disoproxil fumarate; TAF, tenofovir alafenamide; HBV, hepatitis B virus; HCV, hepatitis C virus; PI, protease inhibitor.

Only codes 0 and 1 are accepted. One stop-TAF value of 0.93 and 2 death values of 2 are unknown after cleaning; their meaning was not inferred. Separately, PI exposure in 2 episodes starting after the switch was made missing because its baseline timing could not be established.

Table S2. Inclusive biochemical plausibility bounds and exclusions.

Variable / phase	Lower	Upper	Observed before	Set missing
Creatinine / Before TDF	0.2	15	345	1
Creatinine / TDF phase	0.2	15	341	1
Creatinine / Post-switch TAF phase	0.2	15	241	0
Calcium / TDF phase	4	15	329	5
Calcium / Post-switch TAF phase	4	15	226	5
Phosphorus / TDF phase	0.5	10	330	2
Phosphorus / Post-switch TAF phase	0.5	10	227	1
Vitamin D / TDF phase	0	200	216	0
Vitamin D / Post-switch TAF phase	0	200	207	0

Abbreviations: TDF, tenofovir disoproxil fumarate; TAF, tenofovir alafenamide.

Creatinine, calcium and phosphorus are in mg/dL; vitamin D is ng/mL. A clinically possible extreme within these ranges is retained unless source adjudication justifies correction. A value lying within the bounds is not thereby clinically confirmed.

Table S3A. Cohort reconstruction and primary endpoint availability.

Item	Count or value
Source treatment records	391
Author-confirmed switch cohort	364
Recorded valid switch dates	254
Missing switch dates	110
Paired PCr available	159
Paired PCr unavailable	205
Baseline PCr available	288
Post-switch PCr available	175
Baseline PCr 75th percentile, mg/g	154
Record with anonymous key replacing unavailable source identifier	1
Erroneous birth date masked as missing	1

Abbreviations: PCr, urinary protein-to-creatinine ratio.

Table S3B. Observed baseline comparison and available denominators.

Characteristic	Overall (N = 364)	Paired PCr (n = 159)	No paired PCr (n = 205)	SMD
Age at switch, years	48.46 [41.39, 54.55]; n=254	48.48 [41.40, 55.05]; n=159	48.37 [41.44, 53.82]; n=95	0.12
Time from first HIV contact to switch, years	7.49 [4.20, 14.84]; n=248	7.60 [4.29, 14.97]; n=158	7.04 [3.89, 14.36]; n=90	-0.04
Recorded TDF exposure accrued by switch, years	3.51 [1.78, 6.06]; n=252	3.75 [1.74, 6.23]; n=157	3.27 [1.95, 5.51]; n=95	-0.01
Baseline creatinine, mg/dL	0.88 [0.76, 1.00]; n=340	0.87 [0.78, 0.99]; n=159	0.88 [0.76, 1.01]; n=181	-0.04
Baseline eGFR, mL/min/1.73 m^2	100.76 [89.10, 108.85]; n=238	100.71 [89.65, 108.43]; n=159	100.81 [88.93, 108.95]; n=79	-0.05
Baseline PCr, mg/g	102.00 [70.00, 154.00]; n=288	102.00 [70.00, 146.50]; n=159	100.00 [71.00, 156.00]; n=129	0.06
Baseline calcium, mg/dL	9.34 [9.04, 9.60]; n=324	9.39 [9.09, 9.65]; n=154	9.30 [9.00, 9.59]; n=170	0.21
Baseline phosphorus, mg/dL	3.10 [2.70, 3.50]; n=328	3.05 [2.70, 3.40]; n=157	3.10 [2.73, 3.50]; n=171	-0.04
Baseline vitamin D, ng/mL	24.45 [19.00, 31.77]; n=216	25.00 [19.76, 33.14]; n=117	24.23 [18.80, 31.51]; n=99	0.07
Female sex	101/363 (27.8%)	44/159 (27.7%)	57/204 (27.9%)	-0.01
HBV coinfection	43/348 (12.4%)	19/154 (12.3%)	24/194 (12.4%)	0.00
HCV coinfection	57/357 (16.0%)	22/156 (14.1%)	35/201 (17.4%)	-0.09
PI exposure during TDF	234/358 (65.4%)	107/155 (69.0%)	127/203 (62.6%)	0.14

Characteristic	Overall (N = 364)	Paired PCr (n = 159)	No paired PCr (n = 205)	SMD
Recorded TDF exposure > 96 weeks	260/361 (72.0%)	118/158 (74.7%)	142/203 (70.0%)	0.11

Abbreviations: PCr, urinary protein-to-creatinine ratio; TDF, tenofovir disoproxil fumarate; HIV, human immunodeficiency virus; HBV, hepatitis B virus; HCV, hepatitis C virus; eGFR, estimated glomerular filtration rate; PI, protease inhibitor; SMD, standardised mean difference.

Each numerical cell displays its observed n, so missing count equals its column population minus n. Standardised mean difference (SMD) values are descriptive. Larger missingness in the unpaired group can coexist with small SMDs among the observed values.

Table S3C. Retrospective follow-up indicators.

Indicator	Overall N = 364	Paired PCr n = 159	No pair n = 205
Recorded TAF > 48-week indicator	251/253 (99.2%); unknown 111	158/159 (99.4%); unknown 0	93/94 (98.9%); unknown 111
Stop-TAF indicator	74/252 (29.4%); unknown 112	58/159 (36.5%); unknown 0	16/93 (17.2%); unknown 112
Dropout indicator	80/364 (22.0%); unknown 0	18/159 (11.3%); unknown 0	62/205 (30.2%); unknown 0
Death indicator	17/18 recorded; unknown 346	3/3 recorded; unknown 156	14/15 recorded; unknown 190

Abbreviations: PCr, urinary protein-to-creatinine ratio; TAF, tenofovir alafenamide.

These indicators have no verified temporal relationship to laboratory collection, so they are not baseline predictors or adjudicated reasons for missing PCr. The source renal disease field is entirely empty. Unknown death status must not be treated as survival or used to estimate cohort mortality.

Table S4. Exact PCr sign test sensitivity.

Quantity	Result
Paired observations	159
Decreased / increased / unchanged	85 / 64 / 10
Nonzero differences	149
Proportion decreased among nonzero differences	57.0%
Exact 95% CI for proportion decreased	48.7–65.1%
Exact 2-sided binomial *p*	0.1010

Abbreviations: PCr, urinary protein-to-creatinine ratio; CI, confidence interval.

The sign test excludes ties and tests equal probabilities of decrease and increase. Its $p = 0.1010$ does not show significantly more decreases than increases. This differs from the signed-rank result, which also weights ranks of change magnitude; the latter must not be interpreted as a significant median improvement without the relevant symmetry assumption.

Table S5. Sensitivity to the required PCr reduction.

Required reduction	Events / n	Percent	Wilson 95% CI
10%	87/159	54.7%	47.0–62.3%
20%	99/159	62.3%	54.5–69.4%
30%	112/159	70.4%	62.9–77.0%
50%	137/159	86.2%	79.9–90.7%

Abbreviations: PCr, urinary protein-to-creatinine ratio; CI, confidence interval.

Table S6. Component and composite completeness.

Endpoint	Positive	Negative	Classifiable	Unclassifiable
PCr reduction < 20%	99	60	159	205
Post-switch PCr ≥ 154 mg/g	36	139	175	189
Creatinine increase ≥ 10%	79	147	226	138
Any-positive composite	162	26	188	176

Abbreviations: PCr, urinary protein-to-creatinine ratio.

For the composite, 159 have all 3 components observed: 133 positive and 26 negative. Another 29 are positive on an observed component despite incomplete components; the remaining 176 cannot be classified. Thus 162/188 under the any-positive rule is not interchangeable with 133/159 in the fully observed subset.

Table S7. Observed log(1+PCr) by accrued TDF exposure.

Group	Phase	n	Median [IQR]
TDF ≤ 96 weeks	TDF	41	4.585 [4.263, 5.333]
TDF ≤ 96 weeks	TAF	41	4.466 [4.234, 5.236]
TDF > 96 weeks	TDF	116	4.644 [4.266, 4.914]
TDF > 96 weeks	TAF	116	4.483 [4.159, 4.860]
Duration unavailable	TDF	2	4.749 [4.636, 4.863]
Duration unavailable	TAF	2	4.793 [4.684, 4.902]

Abbreviations: PCr, urinary protein-to-creatinine ratio; TDF, tenofovir disoproxil fumarate; TAF, tenofovir alafenamide; IQR, interquartile range.

The summaries are observed medians and IQRs, not mixed-model estimates. TDF and TAF identify nominal treatment phases with unverified specimen intervals. The unavailable duration group contains only two paired patients and supports no subgroup inference.

Table S8. Exploratory natural-spline checks, n = 155.

HC3 Wald test	χ^2	df	*p*
Joint nonlinearity	1.654	2	0.4373
Overall duration association	1.738	3	0.6285

Abbreviations: HC3, heteroscedasticity-consistent covariance estimator type 3.

The change in log(1+PCr) is regressed on a natural cubic spline of accrued TDF duration (3 degrees of freedom) and the remaining 5 covariates. Internal knots are 827.33 and 1987.67 days, with boundary knots at 28 and 5396 days. Joint nonlinearity tests the 2 added nonlinear directions relative to a linear-duration term; the overall test assesses all 3 duration directions. These unadjusted exploratory tests use HC3 covariance and χ^2 reference distributions. The Figure 1D curve holds age at 48.68 years, baseline log(1+PCr) at 4.635 and eGFR at 100.70, with male sex and no PI exposure. Its intervals are pointwise HC3 t-based 95% intervals for the fitted conditional mean, not simultaneous bands or prediction intervals. The full grid and reference profile are supplied as aggregate CSVs.

Table S9A. Influence of the highlighted creatinine observation.

Quantity	Value
Highlighted baseline creatinine, mg/dL	1.030
Highlighted post-switch creatinine, mg/dL	10.800
Highlighted creatinine change, mg/dL	9.770
Mean creatinine change, all pairs, mg/dL	0.095
Median creatinine change, all pairs, mg/dL	0.030
Mean creatinine change excluding highlighted value, mg/dL	0.052
Median creatinine change excluding highlighted value, mg/dL	0.030
Wilcoxon *p* excluding highlighted value	5.89×10^{-6}

Table S9B. Baseline PCr coefficient in post-switch models.

Analysis	n	Coefficient	HC3 95% CI
Post-switch log(1+PCr), baseline-adjusted	155	0.534	0.379–0.690
exclude max baseline PCr	154	0.564	0.397–0.730
exclude high creatinine case	154	0.535	0.379–0.691

Abbreviations: PCr, urinary protein-to-creatinine ratio; HC3, heteroscedasticity-consistent covariance estimator type 3; CI, confidence interval.

Table S10. Hypothetical decision curve summaries.

Set or reference	Threshold	Net benefit
PCr only	0.20	0.531
PCr only	0.40	0.400
PCr only	0.60	0.204

Set or reference	Threshold	Net benefit
PCr only	0.80	0.044
Without PCr	0.20	0.528
Without PCr	0.40	0.371
Without PCr	0.60	0.057
Without PCr	0.80	0.000
Full predictor set	0.20	0.535
Full predictor set	0.40	0.394
Full predictor set	0.60	0.211
Full predictor set	0.80	0.013
All positive	0.20	0.528
All positive	0.40	0.371
All positive	0.60	0.057
All positive	0.80	-0.887
All negative	0.20	0.000
All negative	0.40	0.000
All negative	0.60	0.000
All negative	0.80	0.000

Abbreviations: PCr, urinary protein-to-creatinine ratio.

For a probability threshold t, a positive prediction is probability ≥ t. Net benefit is TP/n − (FP/n) × t/(1−t), where TP and FP denote true-positive and false-positive classifications, respectively, and n = 159. The full grid runs from 0.05 to 0.80 in steps of 0.01; all-positive and all-negative strategies are reference calculations. Thresholds represent hypothetical weights for false positives relative to true positives. The operational PCr endpoint has no validated associated intervention or clinically established threshold preference, so these curves do not demonstrate clinical utility or justify treatment or monitoring decisions.

Table S11. Held-out permutation importance.

Predictor set	Permuted predictor	Mean AUC decrease	Permutation SD
PCr only	Baseline log(1+PCr)	0.204	0.045
Without PCr	Age at switch	-0.029	0.014
Without PCr	Female sex	0.007	0.020
Without PCr	TDF exposure accrued by switch	-0.029	0.009
Without PCr	PI exposure	0.026	0.019
Without PCr	Baseline eGFR	0.003	0.016

Predictor set	Permuted predictor	Mean AUC decrease	Permutation SD
Full predictor set	Baseline log(1+PCr)	0.195	0.041
Full predictor set	Age at switch	-0.005	0.005
Full predictor set	Female sex	0.003	0.016
Full predictor set	TDF exposure accrued by switch	-0.003	0.005
Full predictor set	PI exposure	0.030	0.019
Full predictor set	Baseline eGFR	-0.007	0.005

Abbreviations: PCr, urinary protein-to-creatinine ratio; TDF, tenofovir disoproxil fumarate; eGFR, estimated glomerular filtration rate; PI, protease inhibitor; SD, standard deviation; AUC, area under the curve.

Each outer model is fitted using its previously selected penalty and training-fold imputation and scaling. One predictor is shuffled within each held-out fold after the fixed training-fold transformations, and the resulting probabilities are pooled to calculate AUC. Thirty repetitions per predictor (seeds recorded in the executable script) give the mean and SD; SD denotes permutation variability, not a confidence interval for population importance. Negative AUC decreases are retained. The reconstructed unpermuted predictions match the finalized held-out probabilities within 3.8×10^{-9}. Importance measures reliance of a fitted predictor set on a variable; it does not establish causality, and baseline-PCr importance is affected by the endpoint definition.
