# Supplementary material for: Assessing Renal Outcomes After Switching from Tenofovir Disoproxil Fumarate to Tenofovir Alafenamide in People with HIV: Proteinuria Changes and Limits of Causal Attribution
Source: Diseases. 2026 Sep 24;14(10):353. doi: 10.3390/diseases14100353 (PMC13703067; doi:10.3390/diseases14100353)
Supplement: Supplementary file 1 [file diseases-14-00353-s001.zip › Supplementary Material S2/Analysis/Figure_S1_ANCOVA_diagnostics.pdf]

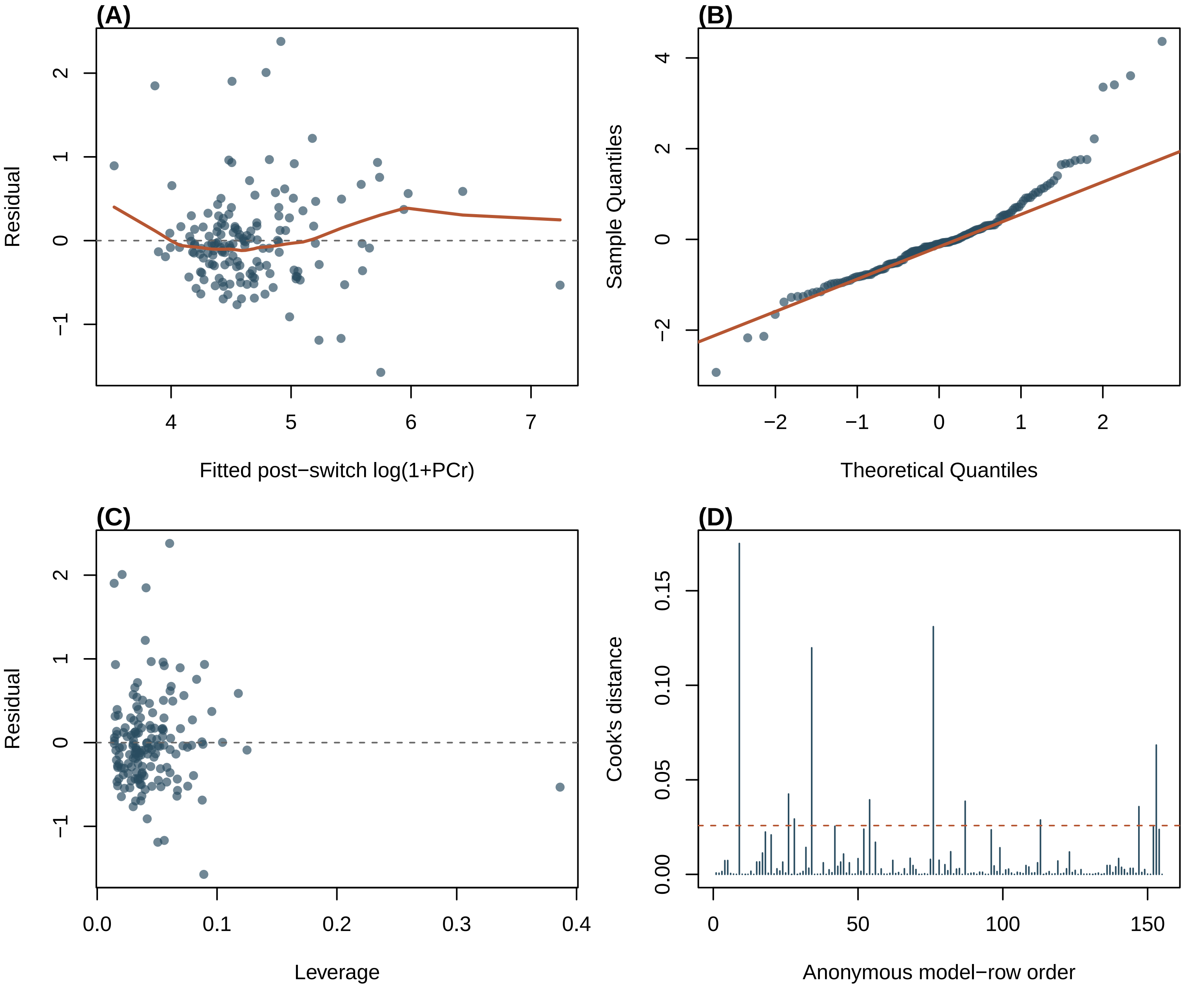
(A)
Residual
Fitted post−switch log(1+PCr)
(B)
Sample Quantiles
Theoretical Quantiles
(C)
Residual
Leverage
(D)
Cook's distance
Anonymous model−row order
