# Supplementary material for: Assessing Renal Outcomes After Switching from Tenofovir Disoproxil Fumarate to Tenofovir Alafenamide in People with HIV: Proteinuria Changes and Limits of Causal Attribution
Source: Diseases. 2026 Sep 24;14(10):353. doi: 10.3390/diseases14100353 (PMC13703067; doi:10.3390/diseases14100353)
Supplement: Supplementary file 1 [file diseases-14-00353-s001.zip › Supplementary Material S2/Figures/Figure_1_PCr_change.pdf]

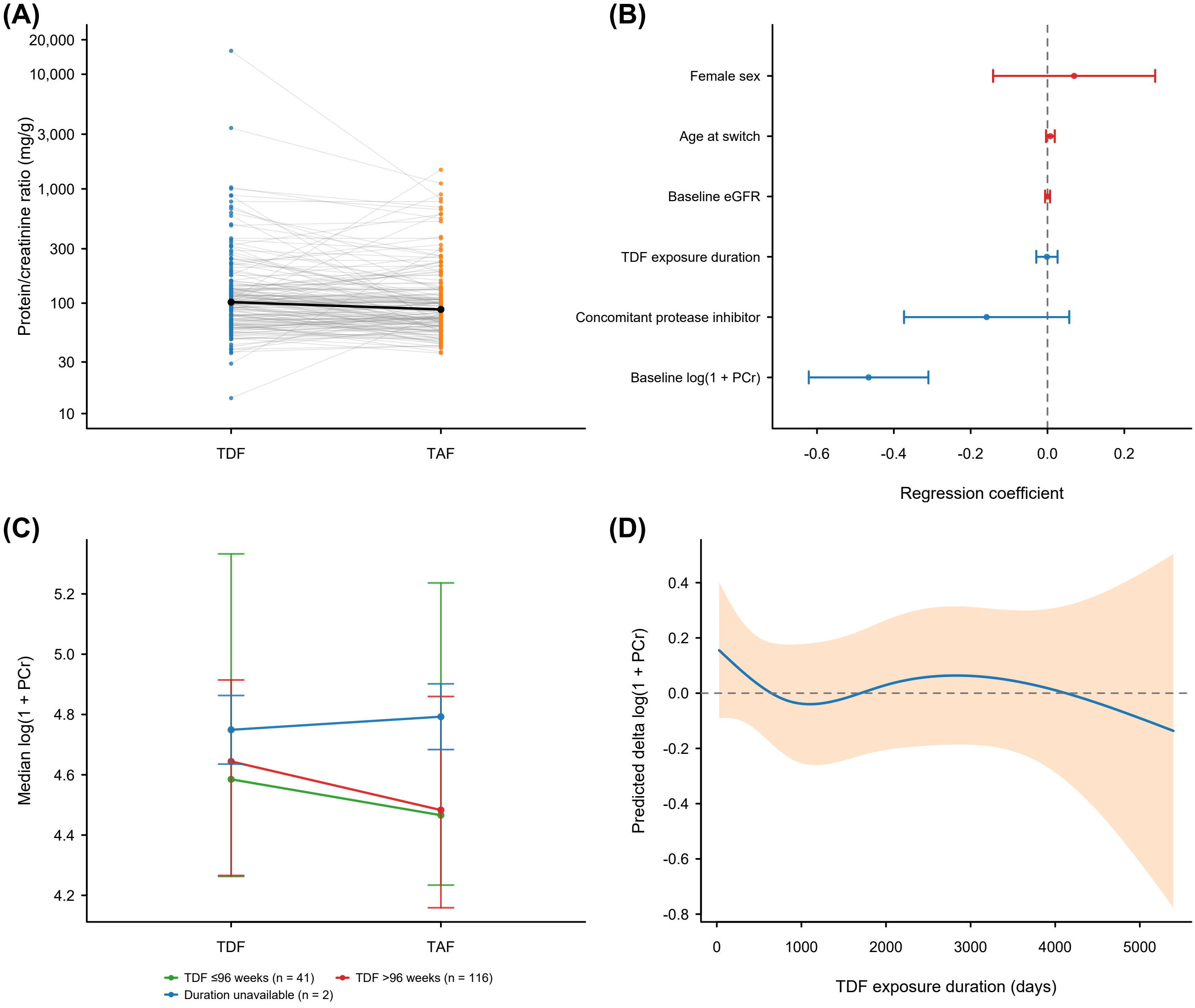
(A)
20,000
10,000
3,000
1,000
300
100
30
10
Protein/creatinine ratio (mg/g)
TDF
TAF
(B)
Female sex
Age at switch
Baseline eGFR
TDF exposure duration
Concomitant protease inhibitor
Baseline log(1 + PCr)
-0.6
-0.4
-0.2
0.0
0.2
Regression coefficient
(C)
5.2
5.0
4.8
4.6
4.4
4.2
Median log(1 + PCr)
TDF
TAF
TDF ≤96 weeks (n = 41)
TDF >96 weeks (n = 116)
Duration unavailable (n = 2)
(D)
0.4
0.2
0.0
-0.2
-0.4
-0.6
-0.8
Predicted delta log(1 + PCr)
0
1000
2000
3000
4000
5000
TDF exposure duration (days)
