# Supplementary material for: Assessing Renal Outcomes After Switching from Tenofovir Disoproxil Fumarate to Tenofovir Alafenamide in People with HIV: Proteinuria Changes and Limits of Causal Attribution
Source: Diseases. 2026 Sep 24;14(10):353. doi: 10.3390/diseases14100353 (PMC13703067; doi:10.3390/diseases14100353)
Supplement: Supplementary file 1 [file diseases-14-00353-s001.zip › Supplementary Material S2/Figures/Figure_2_Biomarker_profiles.pdf]

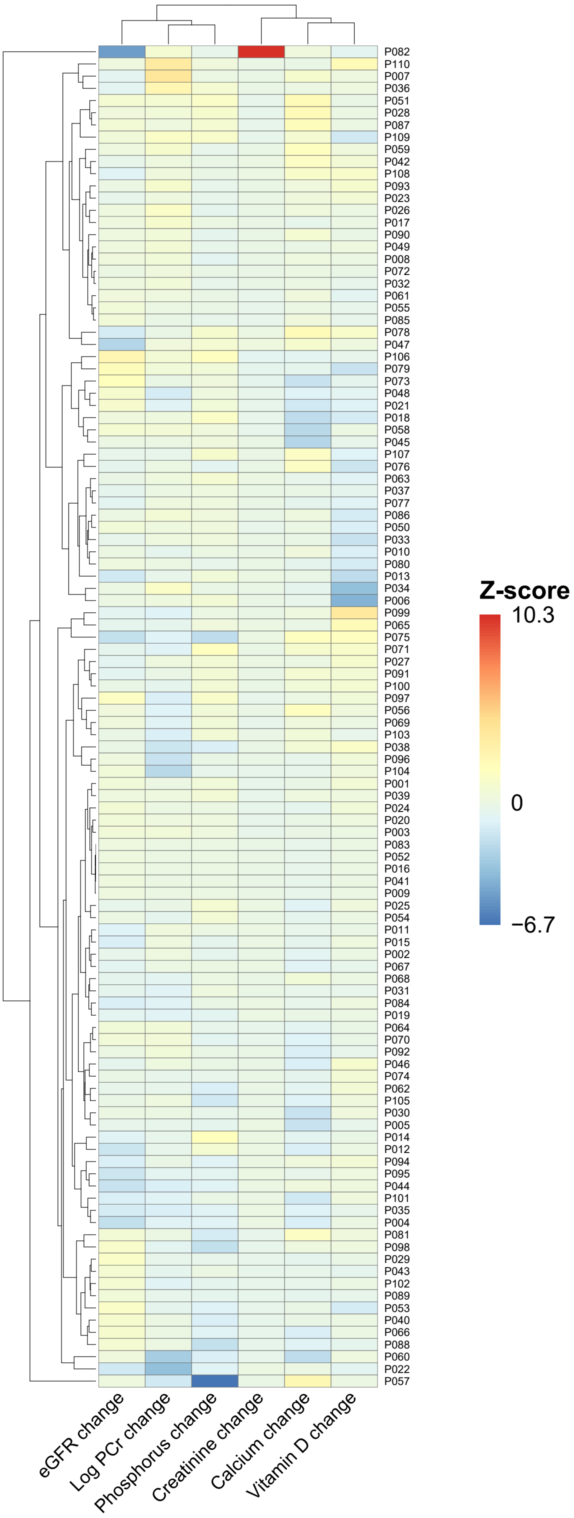
Z-score
10.3
0
−6.7
P082
P110
P007
P036
P051
P028
P087
P109
P059
P042
P108
P093
P023
P026
P017
P090
P049
P008
P072
P032
P061
P055
P085
P078
P047
P106
P079
P073
P048
P021
P018
P058
P045
P107
P076
P063
P037
P077
P086
P050
P033
P010
P080
P013
P034
P006
P099
P065
P075
P071
P027
P091
P100
P097
P056
P069
P103
P038
P096
P104
P001
P039
P024
P020
P003
P083
P052
P016
P041
P009
P025
P054
P011
P015
P002
P067
P068
P031
P084
P019
P064
P070
P092
P046
P074
P062
P105
P030
P005
P014
P012
P094
P095
P044
P101
P035
P004
P081
P098
P029
P043
P102
P089
P053
P040
P066
P088
P060
P022
P057
eGFR change
Log PCr change
Phosphorus change
Creatinine change
Calcium change
Vitamin D change
