# Supplementary material for: Assessing Renal Outcomes After Switching from Tenofovir Disoproxil Fumarate to Tenofovir Alafenamide in People with HIV: Proteinuria Changes and Limits of Causal Attribution
Source: Diseases. 2026 Sep 24;14(10):353. doi: 10.3390/diseases14100353 (PMC13703067; doi:10.3390/diseases14100353)
Supplement: Supplementary file 1 [file diseases-14-00353-s001.zip › Supplementary Material S2/Figures/Figure_3_Nested_CV_calibration.pdf]

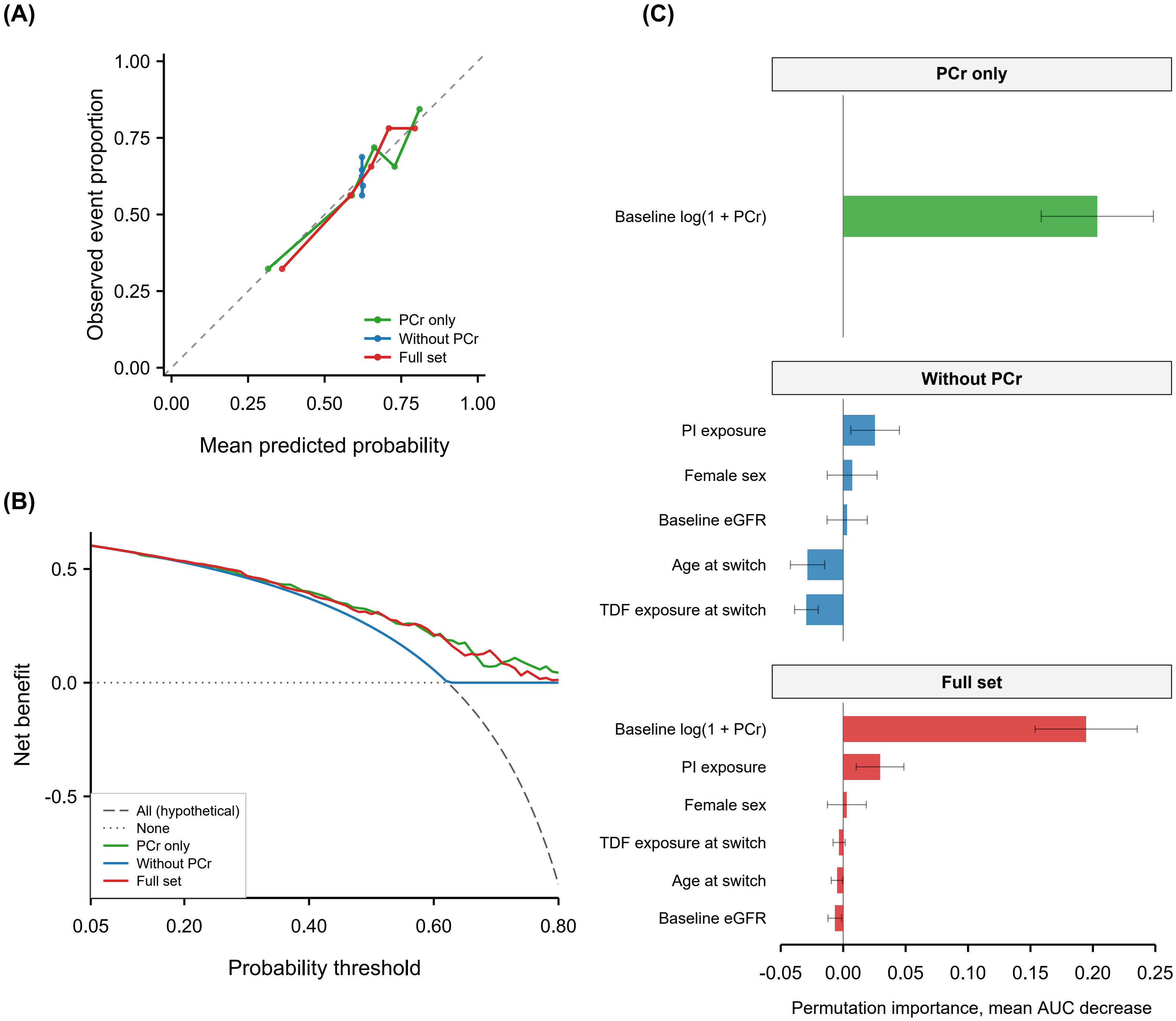
(A)
1.00
0.75
0.50
0.25
0.00
Observed event proportion
0.00
0.25
0.50
0.75
1.00
Mean predicted probability
PCr only
Without PCr
Full set
(B)
0.5
0.0
-0.5
Net benefit
All (hypothetical)
None
PCr only
Without PCr
Full set
0.05
0.20
0.40
0.60
0.80
Probability threshold
(C)
PCr only
Baseline log(1 + PCr)
Without PCr
PI exposure
Female sex
Baseline eGFR
Age at switch
TDF exposure at switch
Full set
Baseline log(1 + PCr)
PI exposure
Female sex
TDF exposure at switch
Age at switch
Baseline eGFR
-0.05
0.00
0.05
0.10
0.15
0.20
0.25
Permutation importance, mean AUC decrease
