# Supplementary material for: Assessing Renal Outcomes After Switching from Tenofovir Disoproxil Fumarate to Tenofovir Alafenamide in People with HIV: Proteinuria Changes and Limits of Causal Attribution
Source: Diseases. 2026 Sep 24;14(10):353. doi: 10.3390/diseases14100353 (PMC13703067; doi:10.3390/diseases14100353)
Supplement: Supplementary file 1 [file diseases-14-00353-s001.zip › Supplementary Material S2/Figures/Figure_4_Nested_CV_discrimination.pdf]

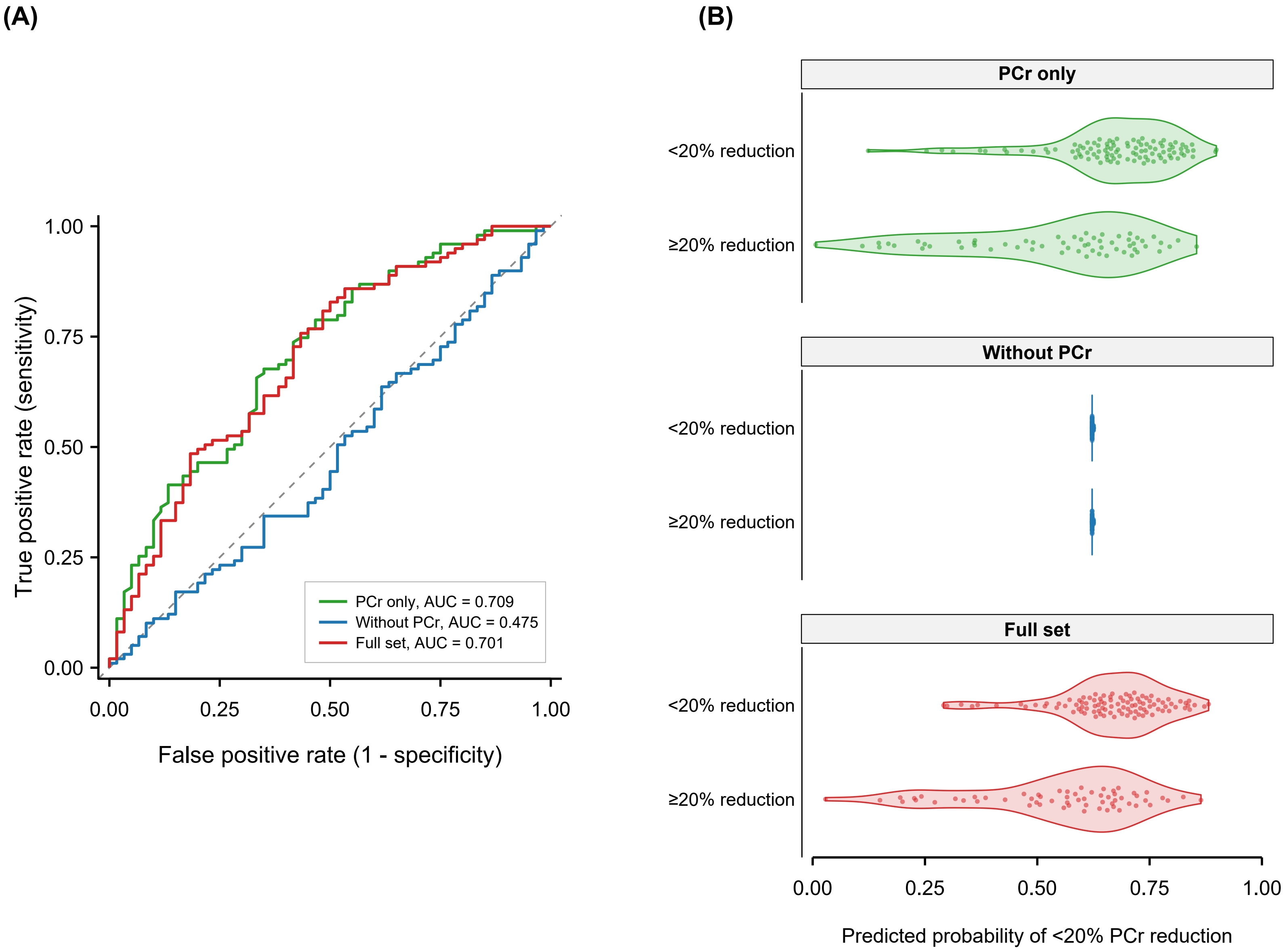
(A)
1.00
0.75
0.50
0.25
0.00
True positive rate (sensitivity)
0.00
0.25
0.50
0.75
1.00
False positive rate (1 - specificity)
PCr only, AUC = 0.709
Without PCr, AUC = 0.475
Full set, AUC = 0.701
(B)
PCr only
<20% reduction
≥20% reduction
Without PCr
<20% reduction
≥20% reduction
Full set
<20% reduction
≥20% reduction
0.00
0.25
0.50
0.75
1.00
Predicted probability of <20% PCr reduction
