# Supplementary material for: Assessing Renal Outcomes After Switching from Tenofovir Disoproxil Fumarate to Tenofovir Alafenamide in People with HIV: Proteinuria Changes and Limits of Causal Attribution
Source: Diseases. 2026 Sep 24;14(10):353. doi: 10.3390/diseases14100353 (PMC13703067; doi:10.3390/diseases14100353)
Supplement: Supplementary file 1 [file diseases-14-00353-s001.zip › Supplementary Material S2/Supplementary informations.pdf]

Supplementary informations

This supplement describes the revised analytical workflow, source-data checks and supporting results. The full cohort comprises 364 author-confirmed switches, with 159 paired urinary protein-to-creatinine ratio (PCr) observations. The analyses characterise an operational proteinuria-change endpoint and do not establish tubular injury, a causal treatment effect or clinical prediction utility.

Supplementary Material S2 contains the de-identified study data, executable analytical and figure code, software information, additional results and reproduction instructions. Tables cited explicitly in the main manuscript are collected separately in Supplementary Material S1. Additional Tables S12–S20 below continue the numbering after the last table cited in the main text.

1. Supplementary methods

Cohort reconstruction. The raw workbook contains 391 treatment rows. Twenty patients contribute repeated rows, giving 27 excess records. Patient-level reconstruction selects the row with the most nonmissing fields; ties retain original source order. Repeated treatment dates and laboratory entries are documented rather than treated as identical duplicate observations. One populated record without a usable source identifier is represented by a unique anonymous key following a check against the other identifying records. The identifier mapping remains restricted. Switch status was confirmed for all 364 reconstructed patients, including 110 without a recorded switch date. One erroneous birth date is masked as missing; no replacement year is inferred.

Timing and predictors. Valid tenofovir alafenamide (TAF) start dates exist for 254 patients. Actual laboratory sampling dates and allowed visit windows are absent. Age at switch, rather than age at data extraction, is used for modelling and at both nominal phases of the Chronic Kidney Disease Epidemiology Collaboration (CKD-EPI) 2021 calculation; the resulting estimated glomerular filtration rate (eGFR) is explicitly approximate. Recorded tenofovir disoproxil fumarate (TDF) exposure accrued by switch is calculated from start to min(stop, switch), expressed in years; 2 negative-duration episodes are missing and 8 later stops are capped. Missing dates do not imply that no switch occurred. The interval from selected TDF stop to TAF start ranges from −1555 to 4721 days (median zero), so immediate replacement cannot be inferred for every source episode. Time from first human immunodeficiency virus (HIV) contact to switch is descriptive only; 6 negative intervals are missing.

Outcome coding. PCr ratios are multiplied by 1000 to mg/g. A relative reduction of less than 20% is event 1 and a reduction of at least 20% event 0; positive baseline and both phase measurements are required. Alternative thresholds were 10%, 30% and 50%. These are descriptive thresholds and do not specify injury severity. The high post-PCr cutoff is the type-7 75th percentile of all 288 available baseline values (154 mg/g). The 3-component composite is positive if any observed component is positive; it is negative only when all components are observed and negative; all other patterns remain missing.

Paired inference. Exactly 6 two-sided Wilcoxon signed-rank p-values are adjusted together using Benjamini–Hochberg: PCr, creatinine, eGFR, calcium, phosphorus and vitamin D. The normal approximation and continuity correction accommodate ties. The test compares signed ranks and does not automatically test the arithmetic mean or median of an asymmetric distribution. Median/interquartile range (IQR) and mean/standard deviation (SD) descriptions therefore answer different summary questions. An exact PCr sign test is a targeted sensitivity analysis. Alpha is 0.05 for nominal exploratory analyses and $q < 0.05$ for the 6-test family; further model coefficients and sensitivity comparisons are not confirmatory family-wise evidence.

Association models. The principal outcome is post-switch log(1+PCr), fitted by ordinary linear regression with baseline log(1+PCr), age at switch, female sex, recorded TDF exposure by switch, protease inhibitor (PI) exposure and baseline eGFR. There are 155 complete cases. Heteroscedasticity-consistent type 3 (HC3) covariance uses squared residuals divided by $(1 - \text{leverage})^2$ in the sandwich estimator; confidence limits use the residual degrees of freedom. The change-score model uses exactly the same rows and predictors. Its baseline-PCr coefficient must be exactly 1 lower, because delta log(1+PCr) equals post minus baseline log(1+PCr); all other coefficients and residuals remain unchanged. This identity is checked in code and prevents treating the 2 models as independent replication. Supporting ordinary logistic regressions at 10%, 20% and 30% use the same complete-case covariates. All converged; they provide exploratory associations, not clinical risk scores. Original high-dimensional model variants were superseded by this focused specification.

Influence and model checks. Residual-versus-fitted and normal-quantile plots, leverage, Cook's distance and the condition number of scaled predictors are supplied in **this archive**. Cook's distance > 4/n and high

leverage are investigation flags rather than automatic deletion rules. The maximum baseline-PCr observation and the highlighted creatinine observation are excluded 1 at a time as sensitivity analyses. Heteroscedasticity-robust intervals do not remove bias from measurement error or unmeasured confounding.

Prediction. One ridge-logistic method compares baseline PCr only, 5 covariates without PCr, and all 6 variables. The objective is mean logistic negative log-likelihood plus λ/2 times the squared standardised non-intercept coefficients. All 3 sets use identical 5 outer stratified folds (seed 4477337); each outer training set has 5 inner stratified folds (seed 6000 + outer-fold number). Fifteen λ values span 10^{-4} to 10^{2}. Mean inner held-out log loss determines λ. Medians for missing predictors and centring/scaling values are fitted afresh in each inner or outer training fold, then applied to its held-out fold. No outcome is imputed. Each of the 159 participants has 1 outer-test prediction per set. Broyden–Fletcher–Goldfarb–Shanno (BFGS) optimisation uses analytic gradients, and the zero-penalty implementation is numerically compared with standard R logistic fitting.

Prediction uncertainty and limits. Area under the receiver operating characteristic curve (AUC) uses average ranks for tied predictions. A total of 2000 patient bootstrap resamples of the fixed out-of-fold predictions (seed 9909) provides percentile AUC intervals and paired AUC differences. These intervals are conditional on the fitted fold procedure and omit full retraining and validation-split uncertainty. The Brier score is mean squared prediction error; log loss evaluates probability accuracy. Calibration intercept uses the logit prediction as an offset; calibration slope estimates its coefficient. Risk quintiles have 31 or 32 participants; Wilson intervals for their observed event rates are supplied in the aggregate calibration table in this archive and are not plotted in Figure 3A. Nearly constant predictions make calibration slope unstable. Decision curves are limited to hypothetical weighted classification; no validated clinical action threshold or deployable final model is claimed.

Missing outcomes. Direct observed-variable comparisons, a date-complete subgroup check, alternative cohort quartiles and logical extreme-case outcome bounds are reported. These do not establish missing-at-random assumptions. Earlier imputation/weighting results are not presented as evidence that selection bias has been eliminated. Follow-up, stop-TAF, dropout and death fields are separately reported with unknown values; blank death fields are not converted to no deaths. Missing binary entries are excluded from their observed denominators and from complete-case association analyses requiring them, while the participant remains in the cohort. Missing predictors in prediction models are imputed within training folds only, as described above; missing outcomes are not imputed. Detailed diagnoses, cluster of differentiation 4 (CD4) cell counts, viral load, HIV type, specimen dates and adjudicated discontinuation reasons are unavailable.

Software and responsibilities. All statistical analyses were personally performed, checked and interpreted by Tommaso D'Anna (T.D.). OpenAI Codex provided ancillary assistance with code refinement, troubleshooting and language editing. T.D. reviewed the analytical code and numerical results and retains responsibility for the analytical decisions and interpretation.

R environment. Analyses used R version 4.6.1 (2026-06-24, Universal C Runtime (UCRT); x86_64-w64-mingw32) on Windows 11, 64-bit, build 26200. The workflow used stats 4.6.1 for statistical tests, regression, numerical optimisation, clustering and density estimation; splines 4.6.1 for natural cubic splines; graphics 4.6.1 for plots; grDevices 4.6.1 for graphics devices, colour handling and Portable Document Format (PDF) and Portable Network Graphics (PNG) export; grid 4.6.1 for figure-panel labels; and utils 4.6.1 for tabular input/output and session information. The recorded execution environment also included base 4.6.1, methods 4.6.1, datasets 4.6.1 and compiler 4.6.1. These are packages distributed as part of base R and share its release version; the example datasets in datasets were not used as study observations. Matrix products used the default R configuration, with Linear Algebra PACKage (LAPACK) 3.12.1. The graphics backends in this R installation were Cairo 1.18.4 and libpng 1.6.54 for the main-figure PDF and PNG exports.

Statistical implementation. Principal functions were stats::wilcox.test, stats::binom.test, stats::p.adjust with method "BH" (Benjamini–Hochberg), stats::lm, stats::glm and stats::optim with method "BFGS"; natural splines used splines::ns. Hierarchical clustering used stats::dist and stats::hclust, and violin distributions used stats::density. HC3 covariance, ridge-logistic fitting, nested cross-validation, bootstrap comparisons, decision curve calculations and held-out permutation importance were implemented in the supplied scripts using these R facilities. The statistical procedures and their assumptions are described above and in Section 3 of this supplement.

Python environment. Source-workbook preprocessing used Python 3.12.14 and openpyxl 3.1.5. The scripts also used pathlib, csv, json, math, statistics, datetime, re, collections, and sys from the Python 3.12.14 standard

library; these modules are versioned with Python. Additional numerical verification was implemented in Python. The reproduction launcher additionally uses argparse and subprocess from the same Python standard library. Package versions not recorded for the analysis were not inferred from other installations.

Verification and provenance. T.D. checked source-data reconstruction, cohort and endpoint counts, missing-value handling, the six-test Benjamini–Hochberg adjustment and the coefficient identity between the post-switch and change-score regressions. Prediction checks addressed training/test separation during preprocessing and tuning, one held-out probability per patient and predictor set, and numerical optimisation; the unpenalised implementation was compared with standard R logistic fitting. Separate Python calculations verified AUC by case-control comparisons, Brier scores, the exact PCr sign test and follow-up counts. This archive contains the executable scripts, input dictionary, recorded session information, random seeds, commands, aggregate results and validation outputs. The de-identified 391-record source workbook, 364-patient numeric reference and analytical dataset, and held-out predictions are supplied in this archive. Names, original identifiers, birth dates, calendar dates and free-text regimens are excluded. Random study keys, within-patient day offsets and age at switch in days retain the quantities needed for numerical reproduction. The original row-selection rule is reproduced using retained fields, the nonmissing count of withheld fields and within-patient tie order; the original analysis order is retained for resampling. The package can be executed without access to the identified source, although the withheld identifying fields cannot be re-audited from it. All 32 regenerated aggregate comma-separated values (CSV) files matched the supplied R1 results, with no numerical differences.

2. Additional results

Table S12. Additional continuous baseline descriptions.

Variable	n	Mean (SD)	Median [IQR]	Range
Age at switch, years	254	47.89 (10.27)	48.46 [41.39, 54.55]	20.76–76.38
TDF exposure by switch, years	252	4.35 (3.50)	3.51 [1.78, 6.06]	0.03–24.00
Baseline creatinine	340	0.95 (0.67)	0.88 [0.76, 1.00]	0.45–9.65
Baseline eGFR	238	98.00 (17.58)	100.76 [89.10, 108.85]	5.43–134.18
Baseline PCr	288	251.34 (1037.54)	102.00 [70.00, 154.00]	14.00–16000.00
Baseline calcium	324	9.31 (0.58)	9.34 [9.04, 9.60]	4.02–10.90
Baseline phosphorus	328	3.17 (0.92)	3.10 [2.70, 3.50]	0.78–9.81
Baseline vitamin D	216	27.01 (12.59)	24.45 [19.00, 31.77]	0.42–80.90

Abbreviations: PCr, urinary protein-to-creatinine ratio; TDF, tenofovir disoproxil fumarate; eGFR, estimated glomerular filtration rate; IQR, interquartile range; SD, standard deviation.

Table S13. Linear model diagnostic summaries.

Diagnostic	Value
Complete cases in linear regression	155.0000
Maximum Cook's distance	0.1750
Observations with Cook's distance > 4/n	10.0000
Maximum leverage	0.3863
Condition number of scaled predictors	2.4817
R^2, post-switch model	0.4468
R^2, change model	0.3463
Spearman correlation, baseline vs post-switch PCr	0.6305
Spearman correlation, baseline vs change in log(1+PCr)	-0.4484

Abbreviations: PCr, urinary protein-to-creatinine ratio.

Table S14. Baseline PCr association under alternative binary thresholds.

Required reduction	n / events	Baseline PCr OR	95% CI	Nominal p
10%	155 / 85	0.284	0.157–0.511	2.76×10^{-5}
20%	155 / 97	0.284	0.160–0.506	1.86×10^{-5}
30%	155 / 110	0.213	0.113–0.403	1.86×10^{-6}

Abbreviations: PCr, urinary protein-to-creatinine ratio; CI, confidence interval; OR, odds ratio.

All 3 ordinary logistic models use the same 6 covariates and 155 complete cases. The odds ratio (OR) is per one-unit increase in baseline log(1+PCr); all models converged. Complete coefficients are included in the accompanying aggregate CSV. The baseline term also enters each endpoint formula, so these associations do not validate a recovery biomarker.

Table S15. Nested cross-validation tuning.

Outer fold	Predictor set	Train / test n	Test events	Selected λ
1	PCr only	127 / 32	20	0.0139
1	Without PCr	127 / 32	20	100.0000
1	Full set	127 / 32	20	0.1000
2	PCr only	127 / 32	20	0.0373
2	Without PCr	127 / 32	20	100.0000
2	Full set	127 / 32	20	0.1000
3	PCr only	127 / 32	20	0.0139
3	Without PCr	127 / 32	20	100.0000

Outer fold	Predictor set	Train / test n	Test events	Selected λ
3	Full set	127 / 32	20	0.0373
4	PCr only	127 / 32	20	0.0139
4	Without PCr	127 / 32	20	100.0000
4	Full set	127 / 32	20	0.0373
5	PCr only	128 / 31	19	0.0052
5	Without PCr	128 / 31	19	100.0000
5	Full set	128 / 31	19	0.0373

Abbreviations: PCr, urinary protein-to-creatinine ratio.

Each penalty was selected on inner held-out log loss, independently of its outer test fold. The 5-fold outer split is identical across predictor sets. Large penalties for the no-PCr set shrink its predictions toward an almost constant event rate.

Table S16. Paired differences in nested cross-validated AUC.

Comparison	AUC difference	Conditional 95% CI
Full minus PCr only	-0.009	-0.056 – 0.039
Without PCr minus PCr only	-0.234	-0.346 – -0.115
Full minus Without PCr	0.225	0.114 – 0.328

Abbreviations: PCr, urinary protein-to-creatinine ratio; CI, confidence interval; AUC, area under the curve.

Table S17. Date-complete and endpoint definition checks.

Check	Value
Patients with recorded switch dates	254.00
Paired PCr among patients with recorded switch dates	159.00
Primary events among patients with recorded switch dates	99.00
Full-cohort baseline PCr quartile, mg/g	154.00
Date-complete baseline PCr quartile, mg/g	151.25
Post-switch PCr events using full-cohort cutoff	36.00
Post-switch PCr events using date-complete cutoff	37.00
Patients with all composite components observed	159.00
Composite events with all components observed	133.00
Composite-positive patients with an unobserved component	29.00
Negative first-contact-to-switch intervals set missing	6.00
TDF episodes starting after switch: duration set missing	2.00

Check	Value
TDF stop dates later than switch: exposure capped	8.00

Abbreviations: PCr, urinary protein-to-creatinine ratio; TDF, tenofovir disoproxil fumarate.

All 159 paired-PCr observations have recorded switch dates; a date-complete analysis therefore leaves the primary event count unchanged while narrowing the descriptive denominator to 254. Under no assumptions for the other 205 primary outcomes, the possible full-cohort event frequency lies between 99/364 (27.2%) and 304/364 (83.5%). These are logical extreme-case bounds, not confidence limits or an estimate of the unobserved outcomes.

Table S18. Extraction time variables retained for description only.

Variable	Overall median [IQR]	Observed n
Age at extraction, years	55.00 [46.00, 61.00]	363
Recorded HIV duration at extraction, years	14.00 [11.00, 21.00]	364

Abbreviations: HIV, human immunodeficiency virus; IQR, interquartile range.

These later-recorded variables are not prediction inputs. The invalid birth-date-derived age is missing. Switch-time age and exposure are used in the revised models.

Table S19. Known and unknown follow-up status.

Indicator	Group	Positive	Negative	Unknown	Total
Recorded TAF > 48-week indicator	Full cohort	251	2	111	364
Recorded TAF > 48-week indicator	Paired PCr	158	1	0	159
Recorded TAF > 48-week indicator	No paired PCr	93	1	111	205
Stop-TAF indicator	Full cohort	74	178	112	364
Stop-TAF indicator	Paired PCr	58	101	0	159
Stop-TAF indicator	No paired PCr	16	77	112	205
Dropout indicator	Full cohort	80	284	0	364
Dropout indicator	Paired PCr	18	141	0	159
Dropout indicator	No paired PCr	62	143	0	205
Death indicator	Full cohort	17	1	346	364
Death indicator	Paired PCr	3	0	156	159
Death indicator	No paired PCr	14	1	190	205

Abbreviations: PCr, urinary protein-to-creatinine ratio; TAF, tenofovir alafenamide.

Nonbinary codes are treated as unknown; missing death status is not survival. These counts describe source recording, without clinical adjudication or verified assessment dates.

Table S20. Logical bounds for the full-cohort primary endpoint.

Quantity	Result
Total cohort	364
Observed events	99
Unobserved primary outcomes	205
Lowest possible cohort frequency	27.2%
Highest possible cohort frequency	83.5%

The lower bound assigns every missing endpoint to no event; the upper assigns every missing endpoint to an event. Neither assignment is claimed to hold, and the interval is not a confidence interval.

3. Supplementary figure analyses

The original coefficient, duration-summary, spline, decision-curve and permutation-importance display types are retained with corrected data and explicit purposes. The additional script reads the finalized analysis data, held-out predictions and selected penalties. It neither repeats penalty selection nor changes the principal results. The same paired sample is used at both phases of the duration-group summary; exposure classes use the selected episode accrued by switch, so post-switch exposure does not enter the grouping.

The duration summaries, spline checks, decision curve summaries and permutation importance tables are provided as Tables S7, S8, S10 and S11 in Supplementary Material S1. Their detailed calculations are implemented in Analysis/conservative_panels_data.R and described in Analysis/README.md.

The R scripts and aggregate outputs in this archive are the numerical source for these supporting tables. Figures use the same reconstructed data and held-out predictions; no original algorithm-ranking values are carried forward.
